# Pathway trajectory analysis with tensor imputation reveals drug-induced single-cell transcriptomic landscape

**DOI:** 10.1038/s43588-022-00352-8

**Published:** 2022-11-24

**Authors:** Michio Iwata, Hiroaki Mutsumine, Yusuke Nakayama, Naomasa Suita, Yoshihiro Yamanishi

**Affiliations:** 1 Department of Bioscience and Bioinformatics, Faculty of Computer Science and Systems Engineering, Kyushu Institute of Technology, Iizuka, Fukuoka Japan; 2 Tsukuba Research Institute, Ono Pharmaceutical Co., Ltd, Tsukuba, Ibaraki Japan

**Keywords:** Drug discovery, Drug development, Computational models

## Abstract

Genome-wide identification of single-cell transcriptomic responses of drugs in various human cells is a challenging issue in medical and pharmaceutical research. Here we present a computational method, tensor-based imputation of gene-expression data at the single-cell level (TIGERS), which reveals the drug-induced single-cell transcriptomic landscape. With this algorithm, we predict missing drug-induced single-cell gene-expression data with tensor imputation, and identify trajectories of regulated pathways considering intercellular heterogeneity. Tensor imputation outperformed existing imputation methods for data completion, and provided cell-type-specific transcriptomic responses for unobserved drugs. For example, TIGERS correctly predicted the cell-type-specific expression of maker genes for pancreatic islets. Pathway trajectory analysis of the imputed gene-expression profiles of all combinations of drugs and human cells identified single-cell-specific drug activities and pathway trajectories that reflect drug-induced changes in pathway regulation. The proposed method is expected to expand our understanding of the single-cell mechanisms of drugs at the pathway level.

## Abstract (web summary)

A method is proposed to predict drug-induced single-cell gene-expression profiles with tensor imputation, detect cell type-specific maker genes, and identify the trajectories of regulated pathways considering intercellular heterogeneity.

## Main

Genome-wide identification of the transcriptomic responses of cells to drug therapies is a promising approach for identifying the mode of action of drugs in medical and pharmaceutical research. Compound-induced transcriptome data have been used to identify their mode of action, including for approved drugs. The Connectivity Map^1^ and the Library of Integrated Network-based Cellular Signatures (LINCS)^2^ have enabled large-scale analysis of drug-induced transcriptome data. However, the transcriptomic response is typically measured in bulk cells, which is a fundamental limitation in understanding the mode of action of drugs at the single-cell level.

Single-cell gene-expression profiling is a powerful tool for understanding cellular systems at the single-cell level^3^. In the field of drug discovery, it is important to understand intercellular heterogeneity to establish efficient therapies^4,5^. The large-scale analysis of drug-induced single-cell transcriptome data is required for a deep understanding of how drugs work at the cellular level; however, such a large-scale single-cell profiling remains costly and difficult. Also, single-cell gene-expression profiling normally generates some degree of missing data due to experimental limitations (for example, difficulty in barcoding cells), which is a major obstacle to their practical application.

Various methods to impute missing values in single-cell gene-expression data have been proposed^6^, most of which are applicable to gene-expression data matrices (for example, for genes and cells). For example, ‘Markov affinity-based graph imputation of cells’ (MAGIC) estimates missing values by sharing information across similar cells^7^. Moreover, ‘single-cell analysis via expression recovery’ (SAVER) considers gene-to-gene relationships to predict missing values^8^. However, these methods cannot be applied directly to gene-expression data that is tensor-structured (for example, for genes, experiments, cells, time points and doses). Indeed, tensor-specific data imputation methods are desired for predicting missing data in single-cell gene-expression profiles^9,10^.

In this Article we present a computational method, ‘tensor-based imputation of gene-expression data at the single-cell level’ (TIGERS), to profile drug activity at the single-cell level. In this method, pathway trajectories that reflect drug-induced changes in pathway regulation are identified by inferring unknown drug-induced single-cell gene-expression data using a tensor imputation algorithm. The proposed method improves the interpretability of single-cell-specific drug activity at the pathway level by considering intercellular heterogeneity.

## Results

### Overview of the method

**Fig. 1:**
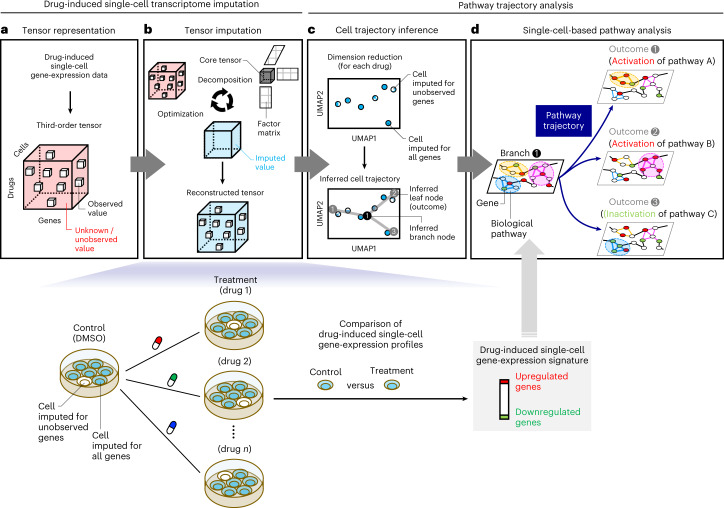
Overview of the proposed method. **a**–**d**, This method consists of two stages: drug-induced single-cell transcriptome imputation (**a**,**b**) and pathway-trajectory analysis (**c**,**d**). **a**, Drug-induced single-cell gene-expression data are represented by a third-order tensor comprising drugs, genes and cells. **b**, Missing or unobserved values in the tensor are comprehensively imputed by tensor decomposition-based optimization. Note that, after tensor imputation, single-cell gene-expression profiles were obtained for all combinations of drugs and cells, and single-cell gene-expression signatures were constructed for all treatment drugs by comparing the corresponding control drug (that is, DMSO). **c**, Cell trajectories are inferred using the imputed drug-induced single-cell gene-expression profiles. **d**, Along the inferred single-cell trajectories, pathway-enrichment analysis is performed, and transient effects of drug activity are identified at the pathway level.

We established a framework for identifying transient drug activity at the pathway level (Fig. 1). First, we represented drug-induced single-cell gene-expression data by a third-order tensor comprising drugs, genes and cells. A third-order tensor was constructed for an appropriate cell group (that is, cell type, cell line or cell lineage; Fig. 1a). For example, in a pancreatic-islet dataset^11^, a benchmark dataset in this study, drug-induced single-cell gene-expression data consisting of four drugs, 23,525 genes and 3,707 alpha cells can be represented by a 4 × 23,525 × 3,707 tensor. However, for a given drug, gene-expression data may not be obtained for all cells. Thus, much of the tensor data is missing or unobserved. Second, we applied a tensor decomposition algorithm, tensor-train decomposition^12^, to the third-order tensor to predict the drug-induced cellular responses in all cells (Fig. 1b). As a result, single-cell gene-expression profiles were obtained for all combinations of drugs and cells, and single-cell gene-expression signatures were constructed for all treatment drugs. Here we term ‘profiles’ as the vectors of gene-expression values measured after drug treatment and ‘signatures’ as the vectors of differences between the gene-expression value measured after drug treatment and those measured in the corresponding control (treated with dimethyl sulfoxide (DMSO)). Thus, after imputation, single-cell-specific drug effects were identified at the pathway level. Third, we applied a dimension-reduction technique to the predicted drug-induced single-cell transcriptome data, where some cells contain observed and imputed gene expression and the other cells contain imputed gene expression only, and inferred cell trajectories along with several coordinates on the two-dimensional map for each drug (Fig. 1c). These coordinates are denoted as vertexes (for example, branch and leaf points), and the inferred cell trajectory corresponds to the cell-state transitions characterized by gene-expression changes. Finally, we identified the impact on pathway regulation for each vertex on the inferred trajectory (Fig. 1d). As a result, we can explain an inferred trajectory from a branch node to a leaf node (outcome) in terms of the impact of a drug on biological pathways. For example, an inferred trajectory from a branch node 1 to a leaf node 1 can be explained by the drug-induced activation of pathway A. Thus, the pathway-trajectory analysis provides information on the transient effects of a drug along the inferred cell trajectory.

### Performance evaluation of the imputation of single-cell data

**Fig. 2:**
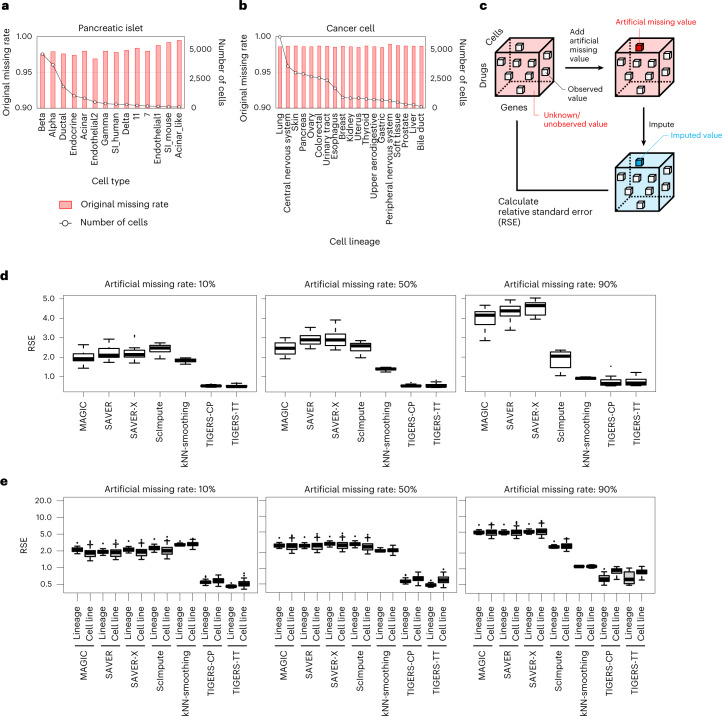
Verification of the imputation accuracy of artificial missing values. **a**, Missing rates for each cell type in the tensor-structured pancreatic-islet dataset. **b**, Missing rates for each cell lineage in the tensor-structured cancer-cell dataset. Cell types or lineages are listed in decreasing order of the number of cells. **c**, Strategy for generating artificial missing values in the tensor-structured drug-induced single-cell gene-expression data. For the artificial missing values, RSEs were calculated between the observed and imputed values. **d**, Performance evaluation of data completion in the pancreatic-islet dataset between seven imputation methods (*n* = 14 cell types). Artificially generated missing rates of 10%, 50% and 90% were tested. **e**, Performance evaluation of data completion in the cancer-cell dataset between seven imputation methods (*n* = 16 for lineages and *n* = 90 for cell lines). Artificially generated missing rates of 10%, 50% and 90% and two different imputation strategies (cell-line-based and lineage-based imputations) were tested. In the box plots: center line, median; box, interquartile range; whiskers, 1.5 × interquartile range; dots, outliers. Source data

In this Article we used two drug-induced single-cell gene-expression datasets (Methods) to evaluate our method. The first is a pancreatic-islet dataset consisting of four drugs, 23,525 genes and 14,368 cells, where each cell has been annotated as one of the 14 cell types^11^. The second is a cancer-cell dataset consisting of nine drugs, 27,342 genes and 31,438 cells, where each cell is from one of 93 cell lines (for example, ACH-000012) in 19 lineages (for example, lung)^13^. Missing ratios in these datasets are more than 95% in each cell group, without regard to the number of cells, suggesting that these datasets have a large number of missing or unobserved values in the third-order tensors (Fig. 2a,b). Note that the definition of the missing ratio is the ratio of missing values to all values in the original gene-expression data.

We evaluated the performance of TIGERS with a tensor-train (TT) decomposition algorithm^12^ to impute missing values in these two datasets. For comparison, we also compared the performance of TIGERS with a previous algorithm, the CANDECOMP/PARAFAC (CP) decomposition algorithm^14^. To evaluate the performance of the imputation, we randomly added artificial missing values to the observed data and tested whether the tensor imputation algorithms could correctly recover these values (Fig. 2c). We defined the artificial missing rate as the ratio of artificially induced missing values against the observed values. As standard imputation methods, we used MAGIC^7^, SAVER^8^, SAVER-X^15^, scImpute^16^ and kNN-smoothing^6^, which are well-established matrix-based imputation methods. We evaluated the relative standard errors (RSEs) between the artificial missing values in the observed data and the imputed values in the reconstructed data.

In the evaluation of the pancreatic-islet dataset, TIGERS performed better than standard imputation methods such as MAGIC and SAVER for all cell types (Fig. 2d and Supplementary Data 1), as the RSEs of TIGERS were smaller than those of standard imputation methods. For example, in the case of artificially generated missing rates of 10%, the RSEs of TIGERS (the mean was 0.527) were significantly smaller than those of standard imputation methods (the mean was 2.136; *P* < 0.01, Wilcoxon rank sum test). The RSEs of all methods tend to become larger in the case of higher missing rates. These results suggest that the tensor imputation algorithms work well for data completion of drug-induced single-cell gene-expression profiles.

For the cancer-cell dataset, we evaluated two imputation strategies, that is, cell-line-based and lineage-based imputations. Missing values were imputed for each cell line in the cell-line-based imputation, but were imputed for each lineage (for example, lung) in the lineage-based imputation. Lineage-based imputation can share the commonality of cell lines in the same lineage. In the evaluation, cell-line-based tensor imputation was difficult for cell lines with higher missing rates (the means of RSEs were 0.553, 0.622 and 0.893 for artificial missing rates of 10%, 50% and 90%, respectively; Fig. 2e and Supplementary Fig. 1). In contrast, lineage-based tensor imputation worked better than cell-line-based imputation for those with any missing rate (*P* values of 0.003, 1.62 × 10^−7^ and 1.05 × 10^−9^ for artificial missing rates of 10%, 50% and 90%, respectively). The other methods show almost similar performance in the two imputation strategies (*P* values of 0.0003, 0.009 and 0.932 for artificial missing rates of 10%, 50% and 90%, respectively). TIGERS with TT decomposition for lineage-based imputation showed the best performance in most cases. Therefore, a consideration of biological commonality, such as clustering cell types by their lineages, on tensor imputation is useful for inferring missing values.

### Biological verification for pancreatic marker genes

**Fig. 3:**
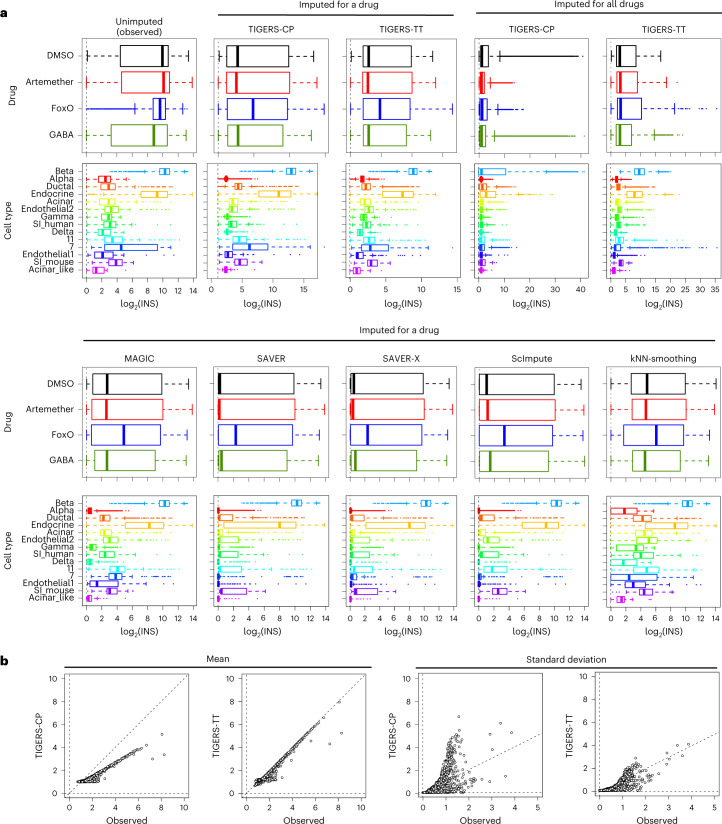
Verification of the imputation ability of missing values. **a**, Distribution of log_2_ expression of insulin (*INS*), a marker gene for beta cells in the pancreatic-islet, with and without imputation. A pseudo count (that is, 1.0) was added to the *INS* expression before log_2_ transformation. In total, 14,368 cells (each treated exclusively with a single drug) and 57,472 profiles (14,368 cells × 4 drugs) were evaluated. In the box plots: center line, median; box, interquartile range; whiskers, 1.5 × interquartile range; dots, outliers. **b**, Comparison of mean and standard deviation of the log_2_ expression of all genes between the unimputed and tensor-based imputed data. The imputed data contain the results of the imputation for all drugs. Source data

In pancreas islets, insulin often serves as a marker for beta cells and glucagon is a marker of alpha cells, because these are specifically and robustly expressed in their respective cell types. We compared the distribution of gene expression before and after imputation of specific marker genes, namely, insulin (*INS*), glucagon (*GCG*), somatostatin (*SST*), pancreatic polypeptide (*PPY*), transthyretin (*TTR*), and regenerating family member 1 alpha (*REG1A*), to evaluate their responses to drugs. For six marker genes in the pancreatic-islet dataset (Methods), cell-type-specific gene expression was preserved after drug treatment (Fig. 3a and Supplementary Fig. 2). For example, *INS* and *GCG* were highly expressed in beta and alpha cells, respectively, as expected. Additionally, for cells imputed for all drugs, TIGERS with TT decomposition could predict cell-type-specific gene expression, whereas the gene-expression levels imputed with CP decomposition were almost random. Furthermore, for all genes, gene expression could be predicted in the range of the observed scale by TT decomposition only (Fig. 3b). These results suggest that TIGERS with TT decomposition can predict potential gene expression for all drugs, even if a cell is not treated with the other drugs.

### Identification of cell-type-specific transcriptome patterns

**Fig. 4:**
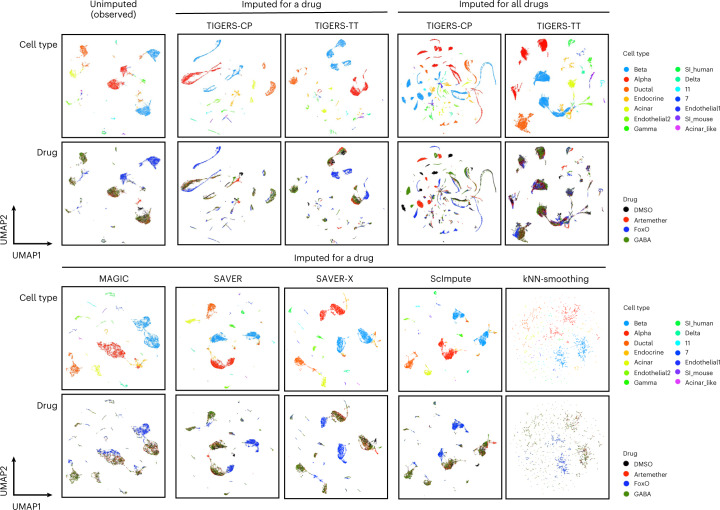
Scatter plots of cells obtained after applying UMAP to gene-expression data with and without imputation. Each profile is colored according to the cell type and the drug in the top and bottom panels, respectively. For cells treated by a drug and those imputed for all drugs, 14,368 cells, each treated by a single drug, and 57,472 (= 14,368 cells × 4 drugs) profiles were evaluated, respectively.

Each cell is represented by a high-dimensional gene-expression profile. To visually identify cell clusters, we reduced the dimensions of the gene-expression data in the pancreatic-islet dataset using the uniform manifold approximation and projection (UMAP)^17^ technique. The gene-expression data were preprocessed using the Monocle3 package^18^ in R (Fig. 4). During application of UMAP to the unimputed data, the missing values were assumed to be zero. On the basis of the UMAP distribution, cells tended to have cell-type-specific gene-expression patterns, which agrees with a study on pancreatic-cell-type-specific gene expression^11^. Both in the observed data containing missing values and by applying matrix-based standard imputation methods (for example, MAGIC and SAVER), almost all cells from the same cell type tended to cluster together; however, cell types did not always separate into clear clusters. In contrast with these standard methods, tensor imputation clearly predicted cell-type-specific gene-expression patterns. For TIGERS with CP decomposition, cell types tended to cluster by drug treatment. Cells from the same cell type that received the same treatment exhibited similar gene-expression patterns, which suggests that TIGERS with CP decomposition predicts different gene-expression patterns for each combination of cell type and drug treatment. In contrast, for TIGERS with TT decomposition, the number of clusters was smaller than that for CP decomposition, and each cluster represented treatment by different drugs. Therefore, TIGERS with TT decomposition did not produce a different cluster for each treatment, which implies that TIGERS-TT might correct the variation explained by a drug while conserving the biological difference between cell types. Taken together, as a result of tensor imputation, cell-specific gene-expression patterns were predicted.

### Characterization of drug activity at the single-cell level

**Fig. 5:**
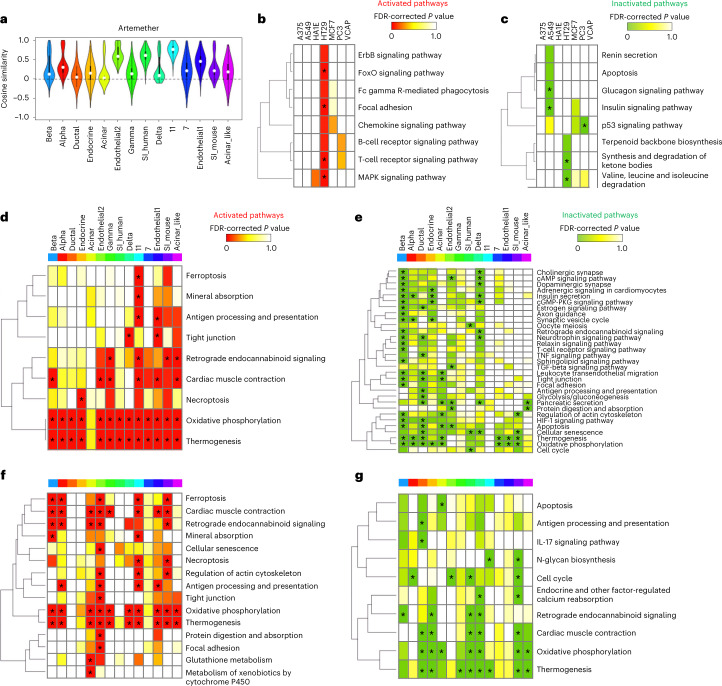
Identification of the mode of action of the antimalarial drug artemether at the single-cell level. **a**, Distribution of cell similarities based on artemether-induced response signatures. **b**, Activated pathways detected using artemether-induced bulk gene-expression data. **c**, Inactivated pathways detected using artemether-induced bulk gene-expression data. **d**, Activated pathways detected using the unimputed artemether-induced single-cell gene-expression data. **e**, Inactivated pathways detected using the unimputed artemether-induced single-cell gene-expression data. **f**, Activated pathways detected using artemether-induced single-cell gene-expression data imputed with TIGERS with TT decomposition. **g**, Inactivated pathways detected using artemether-induced single-cell gene-expression data imputed with TIGERS with TT decomposition. Pathways are listed according to the complete-linkage clustering on the left of each heatmap. Colors in the heatmap correspond to the FDR-corrected *P* values. Significantly enriched pathways are marked with an asterisk. Source data

We constructed drug-induced single-cell response signatures for the pancreatic-islet dataset. The response signatures of the same drug from the same cell type have different response patterns (Fig. 5a and Supplementary Fig. 3), highlighting the importance of the identification of drug activity at the single-cell level. We performed pathway-enrichment analyses to identify biological pathways that are regulated by artemether, an antimalarial drug. Findings from in vivo and in vitro experiments have shown that artemether has anticancer efficacy^19^. Up- and downregulated genes in the artemether-induced response signatures were used to identify activated and inactivated pathways, respectively.

We first assessed the response signatures from bulk tissues, and six out of seven response signatures for artemether treatment were obtained from cancer-cell lines (Fig. 5b,c). For a normal kidney cell line (HA1E) there was no significant gene regulation changes after artemether administration. In contrast, for the cancer-cell lines, several signaling pathways were regulated in a cell-line-specific manner. However, bulk gene-expression data cannot identify pathway regulation at the single-cell level, and the resulting pathways strongly depend on the cell lines used in the bulk response signatures.

These limitations were resolved by using single-cell gene-expression data. Pathways were identified that varied across the cell subsets in pancreatic islets. For the single-cell response signatures (Fig. 5d–g), regulation of oxidative phosphorylation and thermogenesis pathways was detected in almost all cell types, implying that genes on these pathways tended to be regulated by the artemether treatment. To evaluate the effect of imputation, we prepared cell-type-specific gene-expression profiles for all drugs by averaging the single-cell profiles, and constructed cell-type-specific artemether-induced gene-expression signatures. For the unimputed signatures (Fig. 5d,e), significant inactivation of cell-cycle pathways was detected in three cell types, including beta, SI_human and SI_mouse cells. The inactivation of cell-cycle pathways could be relevant for the anticancer activity of artemether. Compared with the unimputed single-cell response signatures, the imputed single-cell response signatures with matrix imputation (Supplementary Fig. 4) showed similar patterns of pathway regulation. For example, the inactivation of cell-cycle pathways was detected in the same cells as those for the unimputed signatures. In contrast, the single-cell response signatures with tensor imputation (Fig. 5f,g) showed a different set of pathways that are potentially regulated by artemether treatment. For example, significant inactivation of cell-cycle pathways in alpha and endotherial2 cells and that of the interleukin-17 (IL-17) signaling pathways in beta and ductal cells were detected after tensor imputation only. It has been reported that the IL-17 family may be involved in cancer development by promoting chronic tissue inflammation^20^. Thus, the inhibitory effect on the signaling pathway might be biologically relevant. This suggests that the proposed tensor imputation method enables the discovery of a different set of biological pathways.

We also performed pathway-enrichment analysis on the FoxOi-induced single-cell gene-expression data (Supplementary Fig. 5). The inactivation of the FoxO signaling pathway was not detected with unimputed data at a significant level. In contrast, after imputation with TIGERS, inactivation was detected in alpha, beta and other cells at a significant level. These results show that the proposed method is useful for understanding the mode of action of drugs.

### Transient drug activity via pathway trajectory analysis

It has been reported that artemether treatment on alpha cells upregulates beta-cell-specific genes and downregulates alpha-cell-specific genes^11^, and the FoxO inhibitor (FoxOi) downregulates alpha-cell-specific genes in alpha cells and beta-cell-specific genes in beta cells^11^. We thus focused on gene-expression data for alpha and beta cells.

**Fig. 6:**
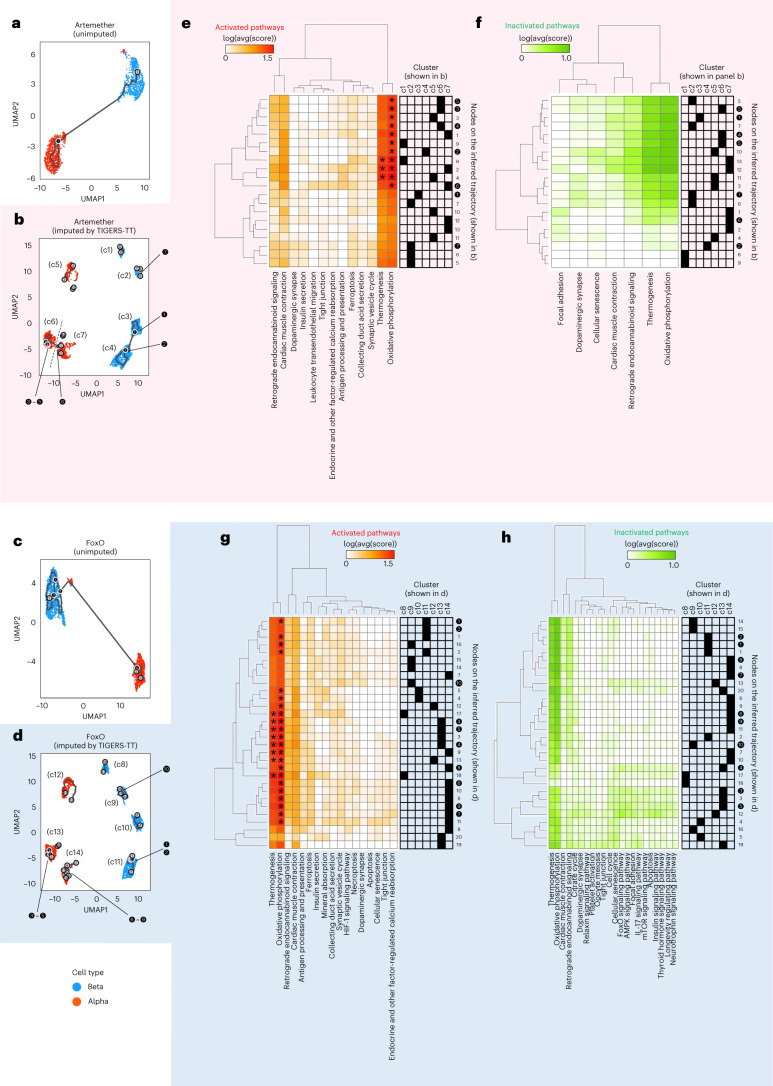
Pathway trajectory analysis. **a**, A trajectory inferred using the unimputed artemether-induced single-cell gene-expression data. The number of cells is 2,184 (1,058 beta and 1,126 alpha cells). **b**, Trajectories inferred using artemether-induced single-cell gene-expression data imputed using TIGERS-TT. The number of cells is 8,327, where 2,184 cells were imputed for unobserved genes and 6,143 cells were imputed for all genes. Indexes of the clusters were manually assigned as cluster numbers c1–c7. **c**, A trajectory inferred using unimputed FoxOi-induced single-cell gene-expression data. The number of cells is 3,157 (1,956 beta and 1,201 alpha cells). **d**, Trajectories inferred using FoxOi-induced single-cell gene-expression data imputed using TIGERS-TT. The number of cells is 8,327, where 3,157 cells were imputed for unobserved genes and 5,170 cells were imputed for all genes. Indexes of the clusters were manually assigned as cluster numbers c8–c14. **e**, Activated biological pathways for each node in **b**. **f**, Inactivated biological pathways for each node in **b**. **g**, Activated biological pathways for each node in **d**. **h**, Inactivated biological pathways for each node in **d**. Trajectories were inferred using Monocle3^18,36^. The black and light-gray circles indicate branch nodes and leaf nodes (different outcomes), respectively. Pathways and nodes are listed according to the complete-linkage clustering. Colors in the heatmap correspond to the logarithmic value of the FDR-corrected *P* value. Black elements in the matrix on the right side of each heatmap indicate the correspondence between cluster numbers and the nodes on the trajectory. Significantly enriched pathways are marked with an asterisk.

We inferred cell trajectories using the unimputed and tensor-based imputed single-cell gene-expression data (Fig. 6a–d). In the unimputed data, each cell was transcriptionally profiled by a drug exclusively; thus, different cells are shown in the scatter plot (Fig. 6a,c). In contrast, after applying tensor imputation, single-cell gene-expression profiles were obtained for all combinations of drugs and cells, resulting in a larger number of cells in the imputed data than in the unimputed data (Fig. 6b,d and Supplementary Fig. 6). The drug-induced gene-expression data in some cells were obtained experimentally, and those in other cells were computationally predicted by tensor imputation. As a result, all cells were transcriptionally profiled for all drugs, and all alpha and beta cells in the dataset are shown in the scatter plot. In the unimputed data, alpha and beta cells were clearly separated into two clusters by the UMAP projection, with each cluster containing several inferred branch points. The two clusters were connected to each other by a trajectory that implies drug-induced cell conversion between alpha and beta cells^11^. In contrast, the tensor-based imputed data included several clusters and a larger number of leaf points, which resulted in unconnected trajectories between different clusters. We analyzed the alpha and beta cells using the Seurat package^21^, and identified alpha-cell-specific marker genes such as *GCG*, heat shock protein family B (small) member 1 (*HSPB1*), coactosin like F-actin binding protein 1 (*COTL1*), vimentin (*VIM*) and nuclear paraspeckle assembly transcript 1 (*NEAT1*), and beta-cell-specific marker genes such as *INS*, spermidine/spermine N1-acetyltransferase 1 (*SAT1*), cytochrome *c* oxidase subunit 5A (*COX5A*), diazepam binding inhibitor, acyl-CoA binding protein (*DBI*) and NPC intracellular cholesterol transporter 2 (*NPC2*) (Supplementary Figs. 7 and 8 and Supplementary Data 2 and 3). In clusters c7 and c14 for the alpha cells, lower expression of *GCG* and higher expressions of *HSPB1*, *COTL1*, *VIM* and *NEAT1* (Supplementary Figs. 9 and 10) were found, and in cluster c4 for beta cells, lower expression of *INS* and higher expressions of *SAT1*, *COX5A*, *DBI* and *NPC2* (Supplementary Fig. 11) were found. Clusters c11 for beta cells and c14 for alpha cells are common in terms of higher expressions of *SAT1*, *COX5A*, *DBI* and *NPC2* (Supplementary Fig. 12). Clusters for the same cell type were located close together, and cells of the alpha and beta types have their own gene-expression patterns. These results show that tensor-based imputation could have the potential to identify not only known marker genes (for example, *INS*), but also new marker genes (for example, *SAT1*), and to infer cell-state transitions in more detail.

Along the inferred trajectories, we performed pathway-enrichment analyses to identify changes in the regulation of biological pathways (Fig. 6e−h). As the trajectory was inferred via the vertexes (for example, branch and leaf points) located nearest to some cells, we predicted pathway regulation for each vertex by averaging the cell-specific pathway regulation patterns. Nodes in the same cluster tended to have similar pathway regulation; however, there were some exceptions. For the example of artemether (Fig. 6e,f), inactivation of the thermogenesis pathway was different among nodes in cluster c7. Also, some nodes in different clusters (for example, c1 and c4) had similar pathway regulation patterns. For FoxOi (Fig. 6g,h), some nodes in clusters 9, 11 and 14 had similar inactivation patterns. Therefore, there may be some transient biological effects among different cell clusters, although the clusters are unconnected by inferred trajectories. Note that, in the unimputed data, because each cell was transcriptionally profiled by a single drug, it is not possible to understand the inferred trajectories in terms of pathway regulation due to the lack of single-cell response signatures. Tensor imputation, on the other hand, provides this possibility.

## Discussion

Recently, a large number of imputation methods have been proposed for predicting missing values in single-cell gene-expression data^6^. Among existing imputation methods, MAGIC^7^ and SAVER^8^ outperformed the other imputation methods most consistently^6^. In this Study, we compared TIGERS with several imputation methods. In matrix-based imputation, missing values are usually imputed only for cells for which gene-expression data are available for at least one drug (or experimental condition), and the gene-expression profile for a cell can be obtained for only a single drug. In contrast, in tensor-based imputation (TIGERS), for all cells in the tensor, drug-induced gene-expression profiles could be imputed. This suggests a notable advantage of tensor-based imputation.

For the pancreatic-islet dataset, we assumed that the cell types assigned to each cell in the previous study^11^ were correct and used the information on these cell types to prepare cell-type-specific tensors. However, it is not always the case that cell types are known for all cells in advance. Unless the information on the cell types is available, the construction of a tensor is difficult in a cell-type-specific manner. In this case, it would be useful to apply unsupervised clustering methods for assigning potential cell types to individual cells.

We performed pathway-enrichment analyses to understand the biological regulation induced by artemether, an antimalarial drug, using bulk and single-cell gene-expression data. Artemether is a derivative of artemisinin, and this family has been recently reported to have antiviral, antiparasitosis, antioxidative, antifibrosis, anti-inflammatory, antidiabetic and anticancer properties^19,22–25^. In our pathway-enrichment analyses, we detected different sets of pathway regulation changes between the bulk and single-cell data. The use of single-cell data enabled to identify drug activities by considering intercellular heterogeneity. Moreover, by using the tensor-based imputed single-cell data, the inactivation of IL-17 signaling pathway was detected in beta and ductal cells, which is a relevant anticancer activity^20^. This case study implies that analyzing the mode of action of drugs at the single-cell level is biologically meaningful.

A limitation of TIGERS is that the imputation performance depends on the missing rate in the original single-cell data. The imputation errors tend to become larger in the case of higher missing rates. TIGERS may not work well for single-cell data with extremely high missing rate in practice. Missing values in single-cell gene-expression data are considered to result from two different scenarios^26^. The first is due to technical limitations on the measurement of gene expression, whereas the second is due to the absence of transcripts in the conditions of interest. However, it is difficult to distinguish between these scenarios in practice. The experimental improvement would contribute to more accurate imputation by TIGERS.

## Methods

### Drug-induced single-cell gene-expression data

We analyzed two drug-induced single-cell gene-expression datasets, one from pancreatic islets^11^ and the other from a panel of cancer cells^13^.

The pancreatic-islet dataset was downloaded from the NCBI Gene Expression Omnibus database (GEO^27^; accession no. GSE142465). The dataset includes raw read counts data from Cell Ranger software (10× Genomics) and an annotation file. The raw counts data were imported using the function Read10X from the Seurat package (v.3.1.5) as unique molecular identifier (UMI) counts. Seurat objects were created as follows: CreateSeuratObject (options: min.cells = 3, min.features = 200). We removed cells for which empty droplets or potential doublets were suspected in the information in the downloaded annotation file. As a result, 93,313 cells were used for downstream analysis. The filtered counts were converted using the function NormalizeData from the Seurat package, with the following parameters: normalization.method = ‘LogNormalize’, scale.factor = 10,000. The study was based on pancreatic islets from three human donors and three mice. The pancreatic islets were subjected to various perturbations for 36 and 72 h. We extracted gene-expression profiles in pancreatic islets from a human donor and four perturbations, namely, DMSO, artemether, the FoxO inhibitor AS1842856 (FoxOi) and GABA, which were measured within 72 h of treatment. The cell type was manually annotated for each cell according to its marker genes, namely, *INS*, *GCG*, *SST*, *PPY*, *TTR* and *REG1A*. The numbers of alpha, beta, gamma and delta cells were 3,707, 4,620, 389 and 313, respectively. In total, 14,368 cells were obtained from this dataset. Note that each cell was treated with one drug exclusively; of 3,707 alpha cells, 741, 1,126, 1,201 and 639 cells were treated with DMSO, artemether, FoxOi and GABA, respectively (Supplementary Table 1).

The cancer-cell dataset was obtained from figshare at https://figshare.com/s/139f64b495dea9d88c70. This study was based on 93 cancer-cell lines from 19 lineages. The cancer cells were subjected to various perturbations at different time points. We extracted gene-expression profiles in one experiment (experiment number 10), in which nine perturbations, namely, DMSO, everolimus, afatinib, taselisib, AZD5591, JQ1, gemcitabine, trametinib and prexasertib were administered, and gene expression was measured within 24 h. The dataset includes raw read counts data from the Cell Ranger software (10× Genomics) and an annotation file. The raw counts data were imported from the Seurat package (v.3.1.5) as UMI counts. Seurat objects were created as follows: CreateSeuratObject min.cells = 0, min.features = 0. We extracted the cells with cell quality = ‘normal’ in the annotation data. The counts were converted using the function NormalizeData from the Seurat package with the following parameters: normalization.method = ‘LogNormalize’, scale.factor = 10,000. The numbers of cells from lung, central nervous system and colorectum were 6,064, 3,565 and 2,589, respectively. In total, 31,438 cells were used from this dataset. Note that each cell was treated with one drug exclusively. For example, of 6,064 cells from lung, 862, 640, 662, 554, 1,112, 810, 293, 510 and 621 cells were treated with DMSO, everolimus, afatinib, taselisib, AZD5591, JQ1, gemcitabine, trametinib and prexasertib, respectively (Supplementary Data 4).

We constructed drug-induced single-cell gene-expression profiles, termed ‘single-cell profiles’. Each single-cell profile was represented by a feature vector ${{{\bf{x}}}} = {\left( {{x_1},\,{x_2},\, \cdots ,\,{x_p}} \right)^{\rm{T}}}$, where *p* is the number of genes (*p* is 23,525 for the pancreatic-islet dataset and 27,342 for the cancer-cell dataset). Each element in the single-cell profile was represented by the gene-expression value measured after drug treatment. Moreover, we constructed drug-induced single-cell gene-expression response signatures, which were termed ‘single-cell response signatures’. Each single-cell response signature was represented by a feature vector ${{{\bf{x}}}}{^\prime} = {\left( {{x^\prime _1},\,{x^\prime _2},\, \cdots ,\,{x^\prime _p}} \right)^{\rm{T}}}$. Each element in the single-cell response signature is the log_2_ ratio of the gene-expression value after drug treatment, *x*^treatment^, to the gene-expression value in the corresponding control (treated with DMSO), *x*^control^. Mathematically, each element is defined as

$${x^\prime _m} = {{{\mathrm{log}}}}_2\frac{{x_m^{{{{\mathrm{treatment}}}}}}}{{x_m^{{{{\mathrm{control}}}}}}}\,\,\,{\left( {m = 1,\,2,\, \ldots ,\,p} \right)}.$$

### Drug-induced bulk gene-expression data

In the Library of Integrated Network-based Cellular Signatures (LINCS) program^2^, gene-expression profiles are obtained based on the L1000 mRNA profiling assay (http://www.lincsproject.org). The gene-expression profiles, namely, GSE70138 and GSE92742, were obtained from the GEO database. These data are based on 93 human cell lines, with various cellular perturbations. The LINCS database provides 978 landmark genes known as the ‘L1000 genes’. In this study we used ‘level 5’ moderated Z-scores (MODZ) data.

The gene-expression levels were measured at 3, 6, 24, 48 and 144 h after drug treatment. Each gene-expression profile (591,855 in total) was represented by a ‘sig_id’. We used 312,596 compound-treatment profiles (denoted as ‘trt_cp’) in total. For each compound, the corresponding International Chemical Identifier code (InChIKey) was also obtained from GEO.

We constructed compound-induced bulk gene-expression response signatures, known as ‘bulk response signatures’. Each bulk response signature was represented by a feature vector ${{{\bf{z}}}} = {\left( {{z_1},\,{z_2}, \cdots ,\,{z_q}} \right)^{\rm{T}}}$, where *q* is the number of L1000 genes (*q* = 978). Each element in the bulk response signature was defined as the difference between the gene-expression value measured after drug treatment and that measured in the corresponding controls (the plate background).

In this study we used bulk gene-expression response signatures for artemether. The response signatures were obtained from seven human cell lines: A375 (malignant melanoma), A549 (lung cancer), HA1E (normal kidney), HT29 (colon cancer), MCF7 (breast cancer), PC3 (prostate cancer) and VCAP (prostate cancer).

### Standard imputation methods for single-cell data

As standard imputation methods, we executed MAGIC^7^, SAVER^8^, SAVER-X^15^, single-cell Impute (scImpute)^16^ and *k*-nearest-neighbor smoothing (kNN-smoothing)^6^ using the Rmagic (version 2.0.3), SAVER (version 1.1.2), SAVER-X (version 1.0.2), and scImpute (version 0.0.9) packages in R (version 4.1.3) and the kNN-smoothing algorithm (version 2.1) in Python (version 3.7.13), respectively. MAGIC was applied to a gene-expression matrix with rows corresponding to cells and columns corresponding to genes, and other methods were applied to a gene-expression matrix with rows corresponding to genes and columns corresponding to cells. All methods were applied to each cell group (that is, cell type, cell line or cell lineage). For example, in the pancreatic-islet dataset, we applied MAGIC to a drug-induced single-cell gene-expression data matrix of 3,707 alpha cells × 23,525 genes. Note that we kept the observed values and replaced the missing entries with predicted values in this study.

### Tensor imputation algorithms for data completion

We evaluated the performance of TIGERS in the prediction of missing values in the drug-induced single-cell gene-expression data. As tensor imputation algorithms, we used TT decomposition and CP decomposition^12,14^. These algorithms handle a tensor with real values, ${{\underline X} \in {\Bbb R}^{I_1 \times I_2 \times \cdots \times I_N}}$, containing missing entries. The variable *I*_*N*_ represents the number of elements in the *N*th mode of a tensor. For example, in a pancreatic-islet dataset, tensor-structured drug-induced single-cell gene-expression data comprising four drugs, 23,525 genes and 3,707 alpha cells has three modes (that is, drugs, genes and cells), where *I*_1_, *I*_2_ and *I*_3_ are 4, 23,525 and 3,707, respectively. The index of the missing entries is recorded by a weight tensor (*W*) with the same size as *X*. Each entry *W* satisfies the conditions as follows:

$${w}_{i_1i_2 \cdots i_N} = \left\{ {\begin{array}{*{20}{c}} {0\,{{{\mathrm{if}}}}\,{x}_{i_1i_2 \cdots i_N}\,{{{\mathrm{is}}}}\,{{{\mathrm{a}}}}\,{{{\mathrm{missing}}}}\,{{{\mathrm{entry,}}}}} \\ {1\,{{{\mathrm{if}}}}\,{x}_{i_1i_2 \cdots i_N}\,{{{\mathrm{is}}}}\,{{{\mathrm{an}}}}\,{{{\mathrm{observed}}}}\,{{{\mathrm{entry}}}}{{{\mathrm{.}}}}} \end{array}} \right.$$

CP decomposition decomposes a tensor into a series of matrices. The CP decomposition of the tensor ${\underline X} \in {\Bbb R}^{I_1 \times I_2 \times \cdots \times I_N}$ can be expressed as

$${\underline X} = {\langle}{\langle}{A}^{(1)},\,{{{{A}}}}^{(2)},\, \cdots ,\,{{{{A}}}}^{(N)}{\rangle}{\rangle},$$

where *A*^(1)^, *A*^(2)^, ..., *A*^(*N*)^ are a series of matrices of sizes *I*_1_ × *R*, *I*_2_ × *R*, ..., *I*_*N*_ × *R*, respectively. *R* is referred to as CP-ranks, which limits the size of each matrix that embeds the latent relationships in the single-cell gene-expression data comprising drugs, genes and cells. We set the CP-rank to 5 because of the grid search for the parameter. Each element of tensor *X* can be written in the following index form:

$${x}_{i_1i_2 \cdots i_N} = {\mathop {\sum }\limits_{r = 1}^R \mathop {\prod }\limits_{n = 1}^N a_{i_nr}^{(n)}},$$

where ${a}_{i_nr}^{(n)}$ is the (*i*_*n*_, *r*)th element of the *n*th matrix.

In the optimization algorithm, the objective variables are the elements of all matrices. Here, the objective function is written as

$${f}{\left( {{{{{A}}}}^{(1)},\,{{{{A}}}}^{(2)},\, \cdots ,\,{{{{A}}}}^{(N)}} \right)} = {\frac{1}{2}||\left( {\underline Y - \underline Z } \right)||^2},$$

where ${\underline Y} = {\underline W \ast \underline X}$ and ${\underline Z} = {\underline W \ast {\langle}{\langle} A^{(1)},\,{{{{A}}}}^{(2)},\, \cdots ,\,{{{{A}}}}^{(N)}}{\rangle}{\rangle}$. The symbol * denotes the Hadamard product^28^.

For *n* = 1, 2, …, *N*, the partial derivatives of the objective function with respect to the *n*th matrix *A*^(*n*)^ can be expressed as

$${\frac{{\partial f}}{{\partial {{{{A}}}}^{(n)}}}} = {\left( {{{{{Z}}}}_{(n)} - {{{{Y}}}}_{(n)}} \right){{{{A}}}}^{( - n)}},$$

where

$${{{{A}}}}^{( - n)} = {{{{A}}}}^{(N)} \odot \cdots \odot {{{{A}}}}^{(n + 1)} \odot {{{{A}}}}^{(n - 1)} \odot \cdots \odot {{{{A}}}}^{(1)}.$$

The symbol ${\odot}$ denotes the Khatri−Rao product^29^.

TT decomposition decomposes a tensor into a series of core tensors, in which all cores are third-order tensors. TT decomposition of the tensor ${\underline X} \in {\Bbb R}^{I_1 \times I_2 \times \cdots \times I_N}$ can be expressed as

$${\underline X} = {\langle}{\langle}{\underline g }^{(1)},\,{\underline g }^{(2)},\, \cdots ,\,{\underline g }^{(N)}{\rangle}{\rangle},$$

where ${\underline g }^{(1)},\,{\underline g }^{(2)},\, \cdots ,\,{\underline g }^{(N)}$ are a series of third-order core tensors of size 1 × *I*_1_ × *r*_1_, *r*_1_ × *I*_2_ × *r*_2_, ..., *r*_*N* − 1_ × *I*_*N*_ × 1, respectively. The sequence {1, *r*_1_, *r*_2_, ..., *r*_*N*__ − 1_, 1} is referred to as TT-ranks, which limits the size of each core tensor. We set the TT-rank to {1, 5, 5, 1} because of the grid search for the parameter. Each element of tensor *X* can be written in the following index form:

$${x}_{i_1i_2 \cdots i_N} = {{{{G}}}}_{i_1}^{(1)} \times {{{{G}}}}_{i_2}^{(2)} \times \cdots \times {{{{G}}}}_{i_N}^{(N)},$$

where ${{{{G}}}}_{i_n}^{(n)}$ is the *i*_*n*_th slice of the *n*th core tensor.

In the optimization algorithm, the objective variables are the elements of all core tensors. Here, the objective function can be written as

$${{f}\left( {\underline g ^{(1)},\,\underline g ^{(2)},\, \cdots ,\,\underline g ^{(N)}} \right) = \frac{1}{2}||\left( {\underline Y - \underline Z } \right)^2}||,$$

where $${\underline Y} = {\underline W \ast \underline X}$$ and $${\underline Z} = {\underline W \ast {\langle}{\langle}\underline g ^{(1)},\,\underline g ^{(2)},\, \cdots ,\,\underline g ^{(N)}}{\rangle}{\rangle}$$.

The relationship between the original tensor and the core tensors can be derived as^30^

$${{{{X}}}}_{(n)} = {{{{G}}}}_{(2)}^{(n)}\left( {{{{{G}}}}_{(1)}^{ > n} \otimes {{{{G}}}}_{(n)}^{ < n}} \right),$$

where for *n* = 1, 2, …, *N*,

$${{{{G}}}}^{ > n} = {\langle \langle \underline g ^{\left( {n + 1} \right)},\,\underline g ^{\left( {n + 2} \right)},\, \cdots ,\,\underline g ^{\left( N \right)}\rangle \rangle \in {\Bbb R}^{R_n \times I_{n + 1} \times \cdots \times I_N}},$$

$${{{{G}}}}^{ < n} = {\langle}{\langle}{\underline g ^{\left( 1 \right)},\,\underline g ^{\left( 2 \right)},\, \cdots ,\,\underline g ^{\left( {n - 1} \right)}\rangle \rangle \in {\Bbb R}^{I_1 \times \cdots \times I_{n - 1} \times R_{n - 1}}},$$

where *G*^>*N*^ = *G*^<1^ = 1 and ${\otimes}$ denotes the Kronecker product^28^. Here, the relationship function *X*_(*n*)_ uses a tensor matricization operation.

For *n* = 1, 2, …, *N*, the partial derivatives of the objective function with respect to the *n*th core tensor ${\underline g }^{(n)}$ can be expressed as follows:

$${\frac{{\partial f}}{{\partial {{{{G}}}}_{(2)}^{(n)}}} = \left( {{{{{Z}}}}_{(n)} - {{{{Y}}}}_{(n)}} \right)\left( {{{{{G}}}}_{(1)}^{ > n} \otimes {{{{G}}}}_{(n)}^{ < n}} \right)^{{{\mathrm{T}}}}}.$$

After the objective function and the derivation of gradient are obtained, the optimization problem can be solved using any of the optimization algorithms based on the gradient descent method^31^. In this study, the maximum iteration number was set to 100 as the stop criterion for optimization.

### Identification of pathway regulation from transcriptome data

We performed pathway-enrichment analyses of up- and downregulated genes using previously reported methods^32,33^. We included 206 biological pathways in the following Kyoto Encyclopedia of Genes and Genomes^34^ categories: metabolism (except for global and overview maps), environmental information processing (except for membrane transport, signaling molecules and interaction), cellular processes (except for transport and catabolism) and organismal systems. In this analysis, we included genes ranked in the top and bottom 5%.

We set *G*_drug_ as denoting a set of up- or downregulated genes in a response signature induced by a drug, and *G*_pathway_ denotes a set of genes in a pathway map. Also, ${r} = {\left| {G_{{{{\mathrm{drug}}}}}} \right|}$, ${k} = {\left| {G_{{{{\mathrm{pathway}}}}}} \right|}$, ${z} = {\left| {G_{{{{\mathrm{drug}}}}} \cap G_{{{{\mathrm{pathway}}}}}} \right|}$, and *l* is the total number of genes in the entire dataset (*l* = 5,340). We assumed that *z* follows a hypergeometric distribution. Thus, the probability of observing an intersection of size *z* between *G*_pathway_ and *G*_drug_ is determined as follows:

$${{P}\left( {G_{{{{\mathrm{pathway}}}}},\,G_{{{{\mathrm{drug}}}}}} \right) = \mathop {\sum }\limits_{i = z}^{{{{\mathrm{min}}}}\left( {k,\,r} \right)} \frac{{\left( {\begin{array}{*{20}{c}} k \\ i \end{array}} \right)\left( {\begin{array}{*{20}{c}} {l - k} \\ {r - i} \end{array}} \right)}}{{\left( {\begin{array}{*{20}{c}} l \\ r \end{array}} \right)}}}.$$

The resulting *P* values were adjusted using the false discovery rate (FDR^35^).

### Pathway trajectory analysis

For the single-cell-based trajectory pathway analysis, we derived the pathways for every single cell with the following procedures. First, we calculated the single-cell response signature for each cell. Each element in the response signature was defined as the difference in the gene-expression value before and after treatment. Second, we selected genes ranked in the top and bottom 5% in the response signature as upregulated and downregulated genes, respectively. Third, we evaluated the probability of observing the interaction of a set of upregulated or downregulated genes in a response signature and a set of genes in a pathway map. Finally, we predicted the pathway regulation for each vertex on the inferred cell trajectory by averaging the cell-specific pathway regulation patterns.

We inferred cell trajectories using the Monocle3 package^18^ in R. Trajectories were inferred via vertexes (for example, branch and leaf points) on the UMAP^17^ projection, where each vertex is regarded as a cell centroid. Then, we predicted the pathway regulation for each vertex by averaging the cell-specific pathway regulation patterns.

We represented an inferred trajectory as ${{{\mathcal{G}}}} = {\left( {v,\,{{\epsilon }}} \right)}$, where *v* is a set of vertexes, *ε* is a set of undirected edges, and the numbers of vertexes and edges are ${\left| v \right|}$ and ${\left| {{\epsilon }} \right|}$, respectively. Because each cell was moved towards its nearest vertex in the inference algorithm, each vertex *v*_*j*_ corresponded to a set of cells. In the pathway-enrichment analysis, we converted a drug-induced single-cell response signature for the *c*th cell that corresponds to vertex *v*_*j*_ to feature vectors ${{{\bf{f}}}}_{{{{{j}}}},\,{{{{c}}}}}^{{{{\rm{act}}}}} =$ ${\left( {f_1^{\,{{{\mathrm{act}}}}},\,f_2^{\,{{{\mathrm{act}}}}},\, \cdots ,\,f_{d_{{{{\mathrm{path}}}}}}^{{{{\mathrm{act}}}}}} \right)^{{{\mathrm{T}}}}}$and ${{{\bf{f}}}}_{{{{{j}}}},\,{{{{c}}}}}^{{{{\rm{inh}}}}} =$ ${\left( {f_1^{\,{{{\mathrm{inh}}}}},\,f_2^{\,{{{\mathrm{inh}}}}},\, \cdots ,\,f_{d_{\,{{{\mathrm{path}}}}}}^{\,{{{\mathrm{inh}}}}}} \right)^{{{\mathrm{T}}}}}$, where ${f_k^{{{{\mathrm{act}}}}}}$ is the negative logarithmic value of the *P* value for pathway activation, ${f_k^{{{{\mathrm{inh}}}}}}$ is the negative logarithmic value of the *P* value for pathway inhibition, and *d*_path_ is the total number of pathways. Therefore, we represented each vertex by feature vectors:

$${{{\bf{f}}}}_{{{{j}}}}^{\,{{{\rm{act}}}}} = {\left( {\overline f _1^{\,{{{\mathrm{act}}}}},\,\overline f _2^{\,{{{\mathrm{act}}}}},\, \cdots ,\,\overline f _{d_{{{{\mathrm{path}}}}}}^{\,{{{\mathrm{act}}}}}} \right)^{{{\mathrm{T}}}}},$$

$${{{\bf{f}}}}_{{{{j}}}}^{\,{{{\rm{inh}}}}} = {\left( {\overline f _1^{\,{{{\mathrm{inh}}}}},\,\overline f _2^{{{{\mathrm{inh}}}}},\, \cdots ,\,\overline f _{d_{{{{\mathrm{path}}}}}}^{\,{{{\mathrm{inh}}}}}} \right)^{{{\mathrm{T}}}}},$$

where ${\overline f _k}$ is the averaged *f*_*k*_ among a set of cells nearest to vertex *v*_*j*_. As a result, the pathway trajectory can be represented by matrices ${{{{F}}}}_{{{{v}}}}^{{{{\rm{act}}}}}$ and ${{{{F}}}}_{{{{v}}}}^{{{{\rm{inh}}}}}$, where rows are vertexes and columns are activated and inactivated pathways, respectively, which presents transient pathway activity along the vertexes on the inferred cell trajectory.

### Coupled bulk RNA-sequencing and single-cell RNA-sequencing dataset

The RNA-seq and single-cell (sc) RNA-seq datasets were downloaded from the NCBI GEO database (accession nos. GSE148465 and GSE149214, respectively). This study was based on the non-small-cell lung carcinoma PC9 cell line. We extracted gene-expression data in the PC9 cell line treated with erlotinib, a receptor tyrosine kinase inhibitor, for 11 days, and those in the untreated cell lines. For the RNA-seq dataset, we applied DESeq package (v.2) to downloaded weight count data (with genes >9 counts) for calculating log_2_-fold changes. For the scRNA-seq dataset, the dataset includes raw read counts data. Seurat objects were created as follows: CreateSeuratObject min.cells = 3, min.features = 200. We used cells for which the number of non-exonic RNA reads was in the range of 300–7,500. The filtered counts were converted using the function NormalizeData from the Seurat package with the following parameters: normalization.method = ‘LogNormalize’, scale.factor = 10,000. In the scRNA-seq dataset, the number of cells was 2,497.

### Reporting summary

Further information on research design is available in the Nature Portfolio Reporting Summary linked to this Article.

### Supplementary information

Supplementary Information Supplementary Results, Discussion, Table 1, Figs. 1–17 and references.

Reporting Summary

Supplementary Data 1 Performance evaluation of data imputation

Supplementary Data 2 List of marker genes for artemether

Supplementary Data 3 List of marker genes for FoxO

Supplementary Data 4 Numbers of cells and drugs in each cell lineage of the cancer-cell dataset

### Source data

Source Data Fig. 2 Numerical source data.

Source Data Fig. 3 Numerical source data.

Source Data Fig. 5 Numerical source data.

## Data Availability

All data are available from public repositories. The pancreatic-islet dataset was downloaded from the NCBI GEO (accession no. GSE142465). The cancer-cell dataset was obtained from figshare at https://figshare.com/s/139f64b495dea9d88c70. Drug-induced bulk gene-expression data were downloaded from the NCBI GEO (accession nos. GSE70138 and GSE92742). The coupled RNA-seq and scRNA-seq datasets were downloaded from the NCBI GEO (accession nos. GSE148465 and GSE149214, respectively). A subset of the data are available from Code Ocean^37^. Source data are provided with this paper. Other source data are available on figshare^38^.

## Code availability

The code can be obtained from Code Ocean^37^.
