# Supplementary material for: Pathway trajectory analysis with tensor imputation reveals drug-induced single-cell transcriptomic landscape
Source: Nat Comput Sci. 2022 Nov 24;2(11):758–70. doi: 10.1038/s43588-022-00352-8 (PMC10768635; doi:10.1038/s43588-022-00352-8)
Supplement: Supplementary file 1 — Supplementary Results, Discussion, Table 1, Figs. 1–17 and references. [file 43588_2022_352_MOESM1_ESM.pdf]

nature computational science

Article

https://doi.org/10.1038/s43588-022-00352-8

Pathway trajectory analysis with tensor imputation reveals drug-induced single-cell transcriptomic landscape

In the format provided by the authors and unedited

Supplementary Information for

Pathway trajectory analysis with tensor imputation reveals drug-induced single-cell transcriptomic landscape

This PDF file includes:

Supplementary Results
Supplementary Discussion
Supplementary Table 1
Supplementary Figures 1 to 17
Supplementary References

Other Supplementary Materials for this manuscript includes:

Supplementary Data 1 to 4 as xlsx files
Supplementary Data 5 as a zip file (available on figshare)

Supplementary Results

Characterization of the activity of an anticancer drug at the single-cell level

We performed analyses to discuss pathways regulated by afatinib, an anticancer drug in the cancer dataset in this study. **Supplementary Figure 13** shows the heat maps of the regulated pathways detected from afatinib-induced single-cell gene expression data. Several cancer-related pathways, such as the p53 signaling pathway, cell cycle pathway, and apoptosis pathway, were detected in various cancer tissues. For example, the activation of the apoptotic pathway was significantly detected only after imputation, which suggests that the imputation could recover the biologically relevant mode-of-action of anticancer drugs. Note that the same observation was not obtained from the other methods.

Comparisons between second-order and third-order tensor imputations

We compared different imputation methods in the context of the second-order tensor imputation. **Supplementary Figure 14** shows the distribution of relative standard errors (RSEs) between the artificial missing values in the observed data and the imputed values in the reconstructed data. The tendency of performance of second-order tensor imputation methods is similar to that of third-order tensor imputation methods.

Biological verification of imputed values for a lowly expressed gene

We evaluated the expression of T-cell surface glycoprotein CD4 molecule (CD4) as an example of lowly expressed genes. **Supplementary Figure 15** shows the distribution of log2 expression of CD4 with and without imputation. In the unimputed data, the log2 expression of CD4 is between 1.0−1.2, which is much lower than that of INS (**Fig. 3a**). For missing entries, the standard imputation methods produced zero values, whereas TIGERS produced certain values within the range of 1.0−1.5. These results show TIGERS can predict the potential gene expression values close to the observed expression values in the unimputed data.

Correlations between bulk RNA-seq and single-cell RNA-seq dataset with and without imputation

We evaluated the correlation of the imputed missing values by TIGERS in the coupled RNA-seq and scRNA-seq datasets. We imputed the missing values in the scRNA-seq dataset and evaluated the correlations between the imputed values and gene expression values in the RNA-seq dataset. **Supplementary Figure 16** shows the correlations between the erlotinib-induced bulk RNA-seq data and the erlotinib-induced single-cell RNA-seq data with and without imputation. Unimputed (observed) single-cell RNA-seq

data have moderate correlations with the bulk RNA-seq data (the cosine coefficient is 0.432), and similar correlations are observed using single-cell data imputed by MAGIC (0.462), SAVER (0.439), SAVER-X (0.443), and kNN-smoothing (0.382). The single-cell data imputed for a drug by TIGERS with TT decomposition has some correlations (0.284). These results show that the gene expression pattern predicted by TIGERS is not always correlated with the bulk gene expression pattern.

Evaluation of the robustness of the proposed method on technical replicates

To evaluate the robustness of the proposed method on technical replicates, we subsetted the gamma cells from the pancreatic dataset, imputed the missing entries independently and evaluated the imputation performance. First, the gene expression data consisting of 4 drugs, 23,525 genes, and 389 gamma cells were divided into three subsets. Each subset was represented by 4 × 23,525 × 130, 4 × 23,525 × 130, and 4 × 23,525 × 129 tensors. Then, the missing entries in each tensor were imputed by TIGERS. Finally, pathway enrichment analysis was performed using artemether-induced gene expression signatures calculated from each subset. **Supplementary Figure 17** shows Venn diagrams comparing the numbers of activated and inactivated pathways detected using each subset. Some pathways were identified from all three subsets, supporting the

robustness of the proposed method.

Supplementary Discussion

In the past, large-scale drug-induced gene expression data in bulk cell lines[1,2] were utilized for a variety of applications in drug discovery. Thus, large-scale profiling drug responses at the single-cell level would be highly useful, but missing gene expressions are an obstacle in practice. TIGERS is the first method for predicting missing gene expressions for all combinations of drugs and cells and is expected to be widely used in the drug mode of action analysis at the single-cell level toward precision medicine.

Our objectives in this study are to impute missing elements in single-cell data and to reveal the trajectory of drug-induced pathways at the single-cell level. The datasets in this study are not involved in individuals with diseases. Our method could be used for imputing missing values in single-cell data of disease individuals, and the imputed data (completed data) could be analyzed using any stratification methods.

Supplementary Table

Supplementary Table 1 | Numbers of cells and drugs in each cell type of the pancreatic islet dataset. Cell types were manually annotated in a previous study[3].

Cell type	Number of cells	Number of drugs			
		DMSO	Artemether	FoxOi	GABA
Beta	4,620	913	1,058	1,956	693
Alpha	3,707	741	1,126	1,201	639
Ductal	1,847	481	382	274	710
Endocrine	1,046	230	312	266	238
Acinar	844	229	238	116	261
Endothelial2	501	86	157	158	100
Gamma	389	82	121	126	60
SI_human	338	111	62	87	78
Delta	313	77	72	79	85
11	220	41	67	42	70
7	181	32	14	114	21
Endothelial1	155	37	23	79	16
SI_mouse	107	28	31	24	24
Acinar_like	100	31	11	22	36

Supplementary Figures

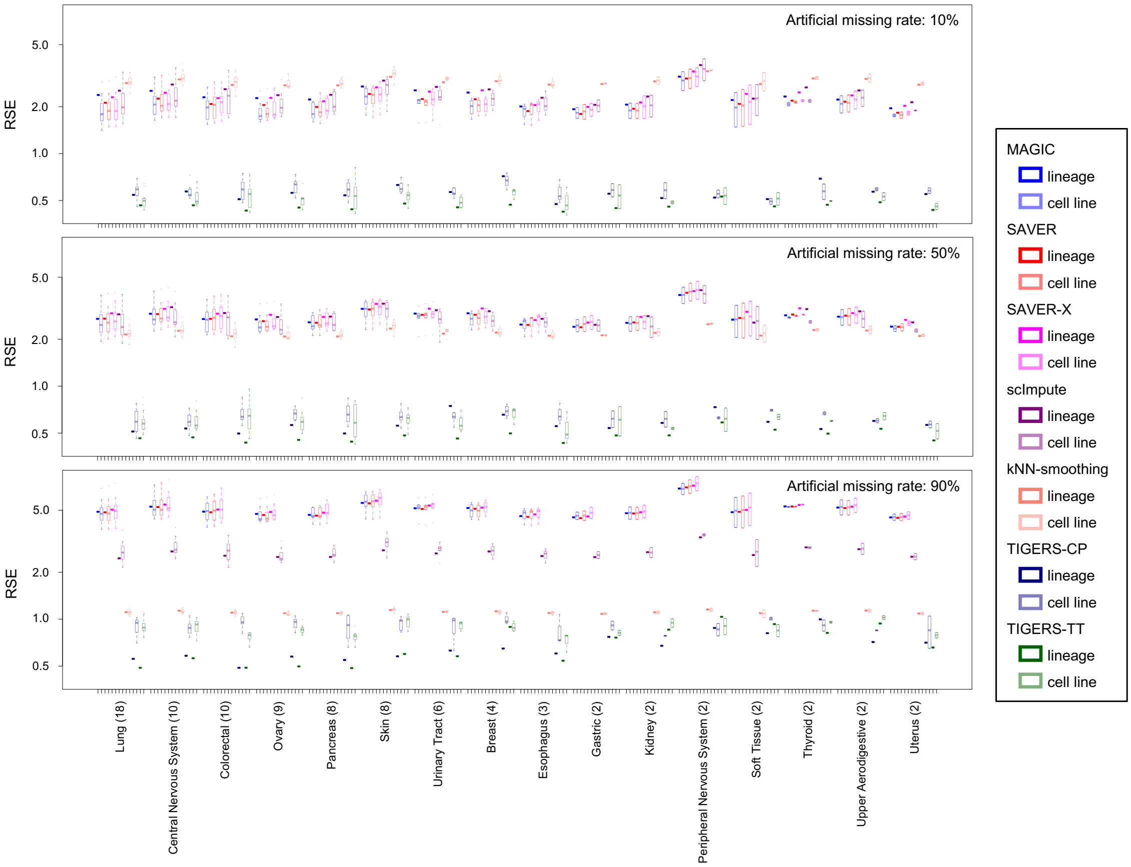

Supplementary Figure 1 | Performance evaluation of data completion in the cancer cell dataset between seven imputation methods. Artificially generated missing rates of 10%, 50%, and 90% and two different imputation strategies (i.e., cell line-based and lineage-based imputations) were tested. Cell lineages are listed in decreasing order of the number of cell lines in the lineage (shown in brackets).

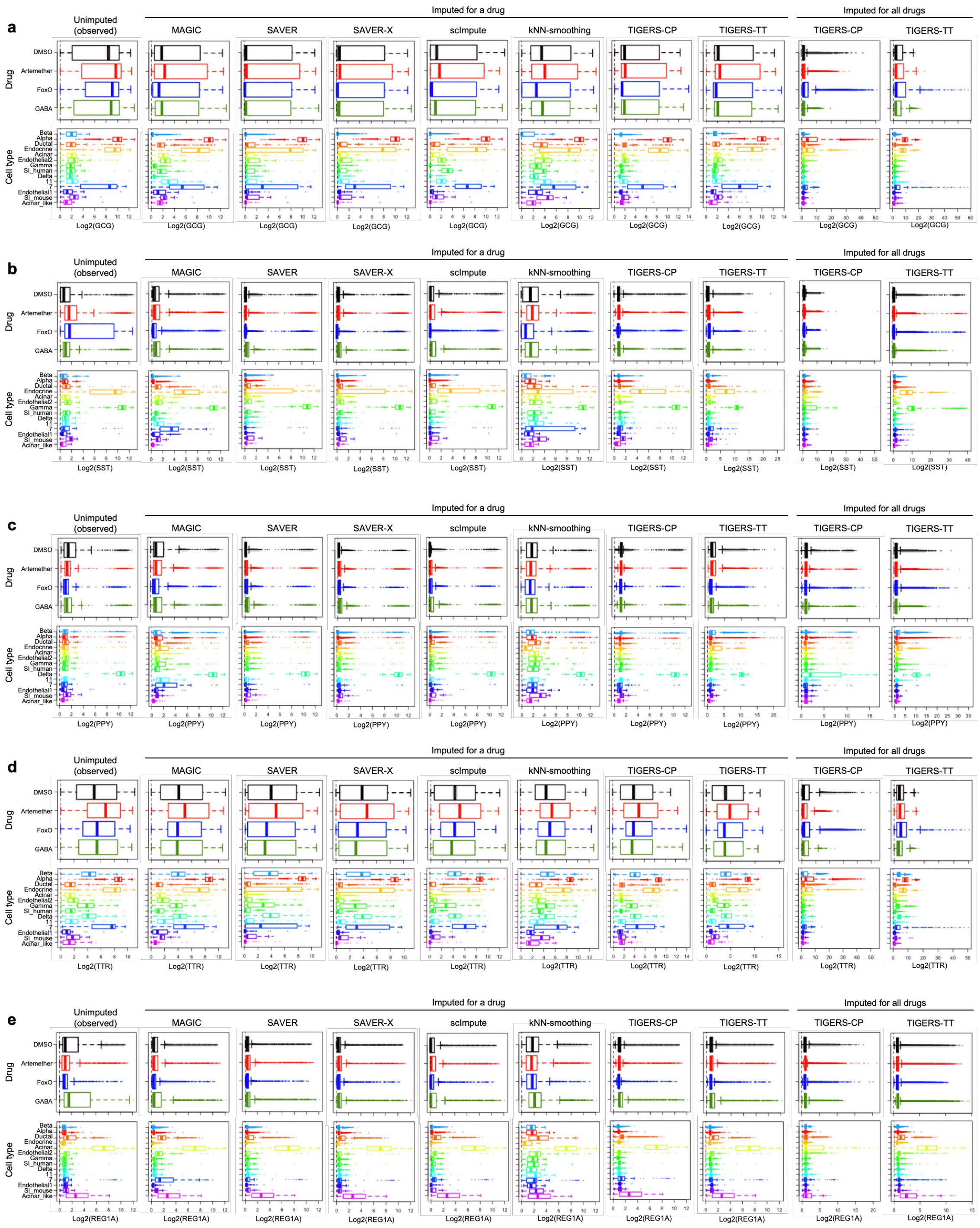

Supplementary Figure 2 | Density plots of log2 expression of marker genes; i.e., **(a)** *GCG*, **(b)** *SST*, **(c)** *PPY*, **(d)** *TTR*, and **(e)** *REG1A*, with and without imputation. Each curve is colored according to the cell type and the drug in the top and bottom panels,

respectively. For cells treated by a drug and those imputed for all drugs, 14,368 cells, each treated by a single drug, and 57,472 (= 14,368 cells × 4 drugs) profiles were evaluated, respectively. In the box plots: center line, median; box, interquartile range; whiskers, 1.5 × interquartile range; dots, outliers.

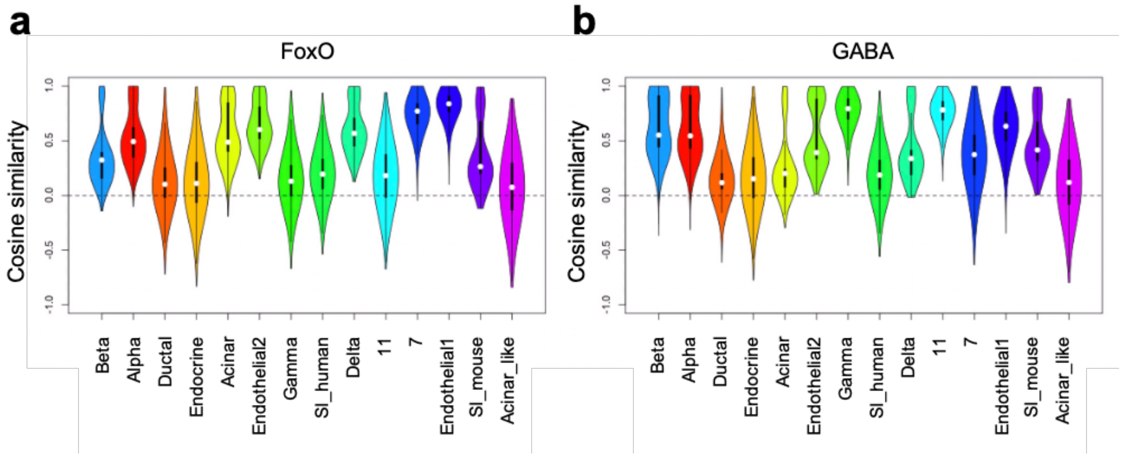

Supplementary Figure 3 | Distribution of cell similarities based on drug-induced response signatures. **(a)** Distribution for FoxOi-induced response signatures. **(b)** Distribution for GABA-induced response signatures. Cell types are listed in decreasing order of the number of cells. In the box plots: center line, median; box, interquartile range; whiskers, 1.5 × interquartile range; dots, outliers.

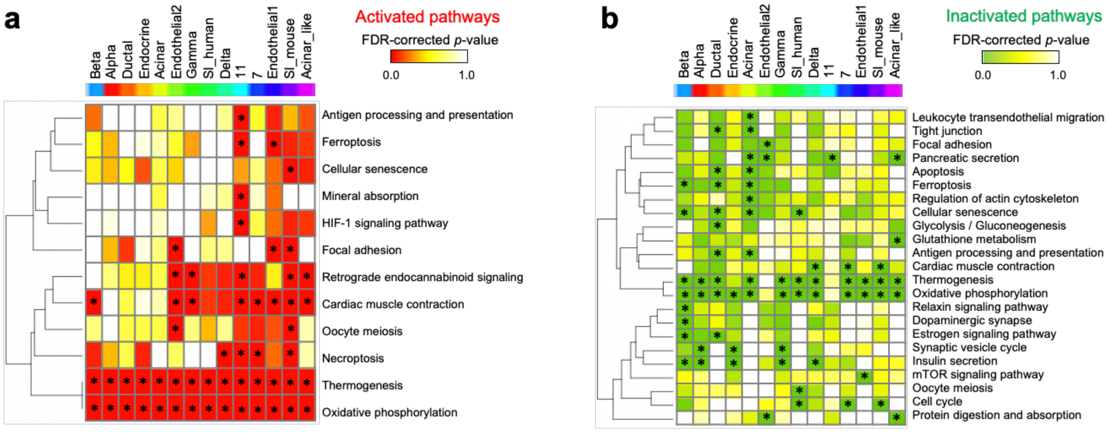

Supplementary Figure 4 | Regulated pathways detected using artemether-induced single-cell gene expression data imputed with MAGIC. **(a)** Activated pathways. **(b)** Inactivated pathways. Pathways are listed according to the complete-linkage clustering on the left of each heatmap. Colors in the heatmap correspond to the FDR-corrected *p* values. Significantly enriched pathways are marked with an asterisk.

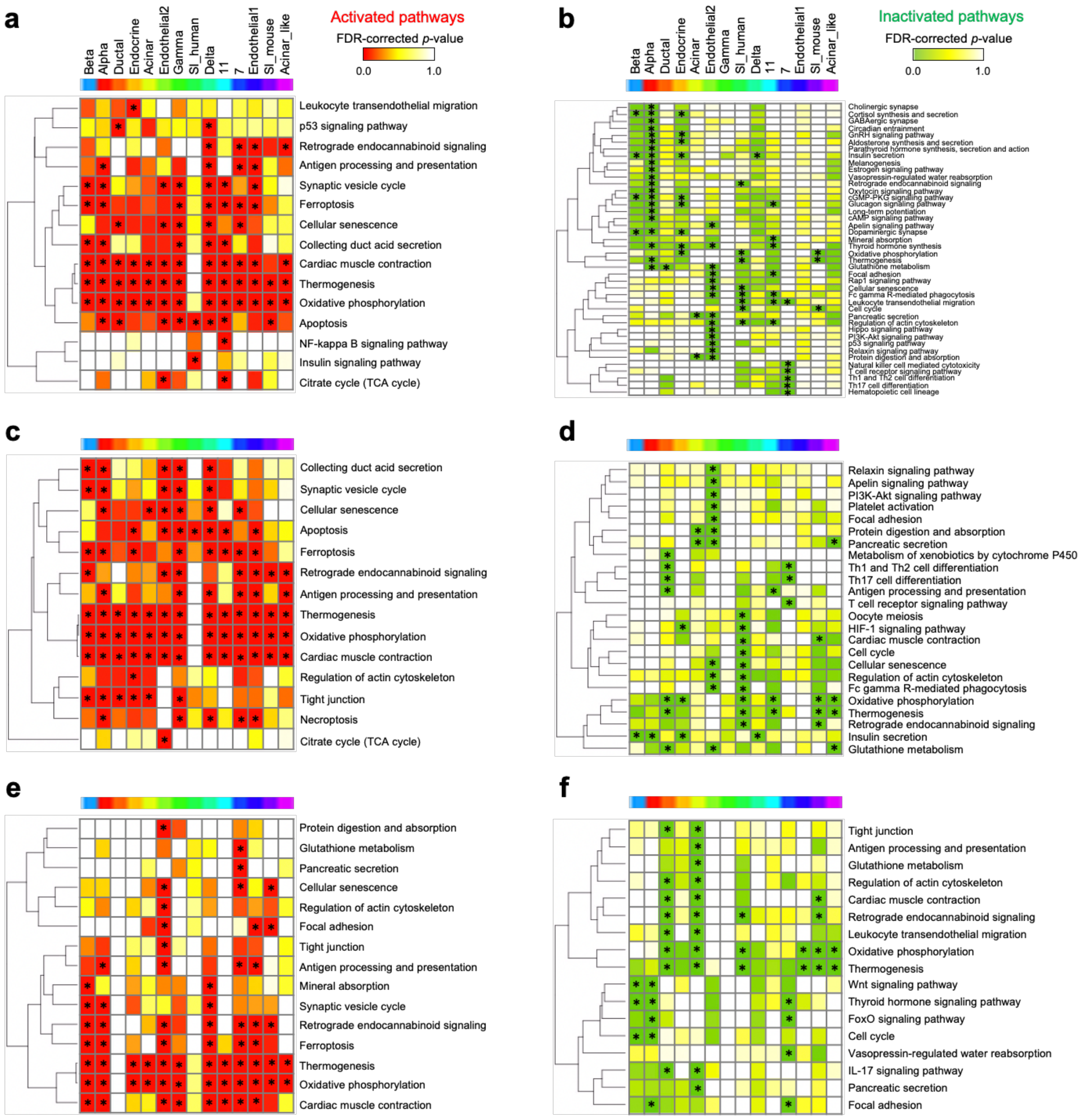

Supplementary Figure 5 | Identification of the mode of action of FoxO at the single-cell level. **(a)** Activated pathways detected using the unimputed FoxO-induced single-cell gene expression data. **(b)** Inactivated pathways detected using the unimputed FoxO-induced single-cell gene expression data. **(c)** Activated pathways detected using FoxO-induced single-cell gene expression data imputed with MAGIC. **(d)** Inactivated pathways detected using FoxO-induced single-cell gene expression data imputed with

MAGIC. **(e)** Activated pathways detected using FoxO-induced single-cell gene expression data imputed with TIGERS with TT decomposition. **(f)** Inactivated pathways detected using FoxO-induced single-cell gene expression data imputed with TIGERS with TT decomposition. Pathways are listed according to the complete-linkage clustering on the left of each heatmap. Colors in the heatmap correspond to the FDR-corrected *p* values. Significantly enriched pathways are marked with an asterisk.

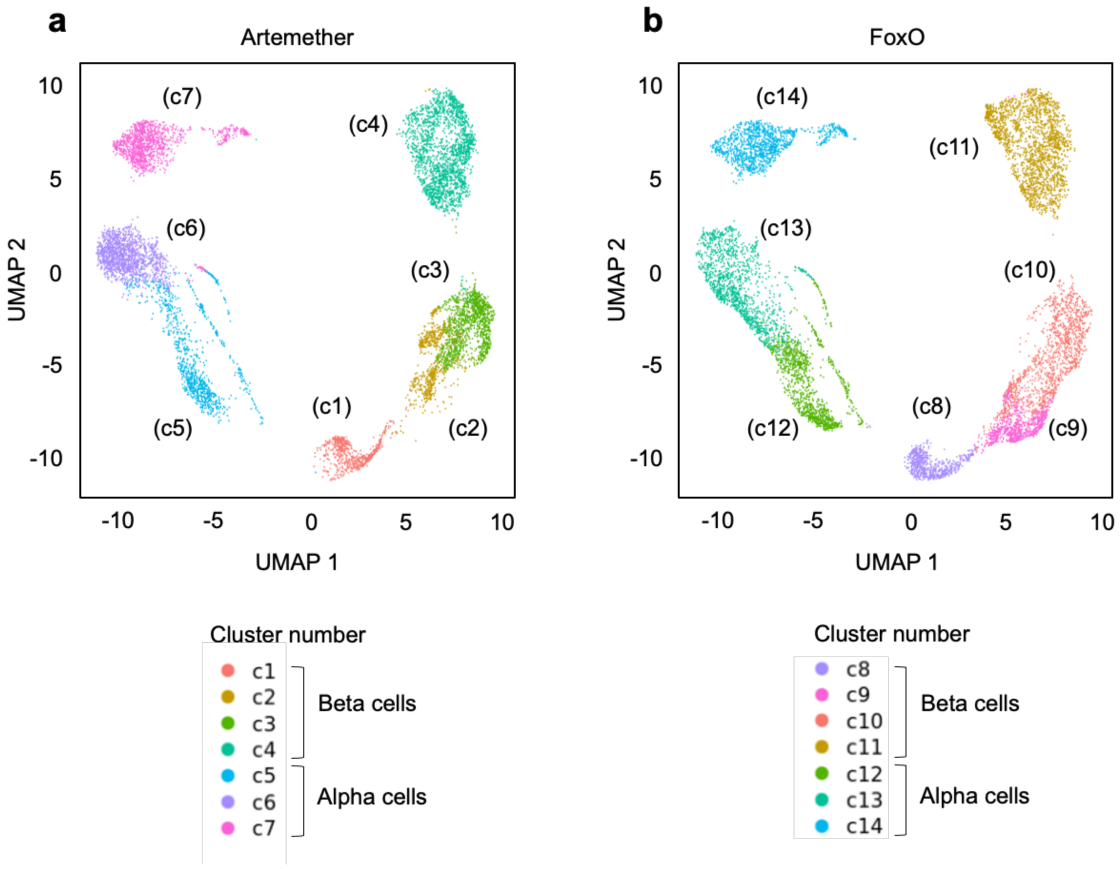

Supplementary Figure 6 | Scatter plots of alpha and beta cells obtained after applying Uniform Manifold Approximation and Projection (UMAP) to gene expression data imputed by TIGERS with TT decomposition using the Seurat package[4]. Each cell is colored according to the cluster numbers c1–c14.

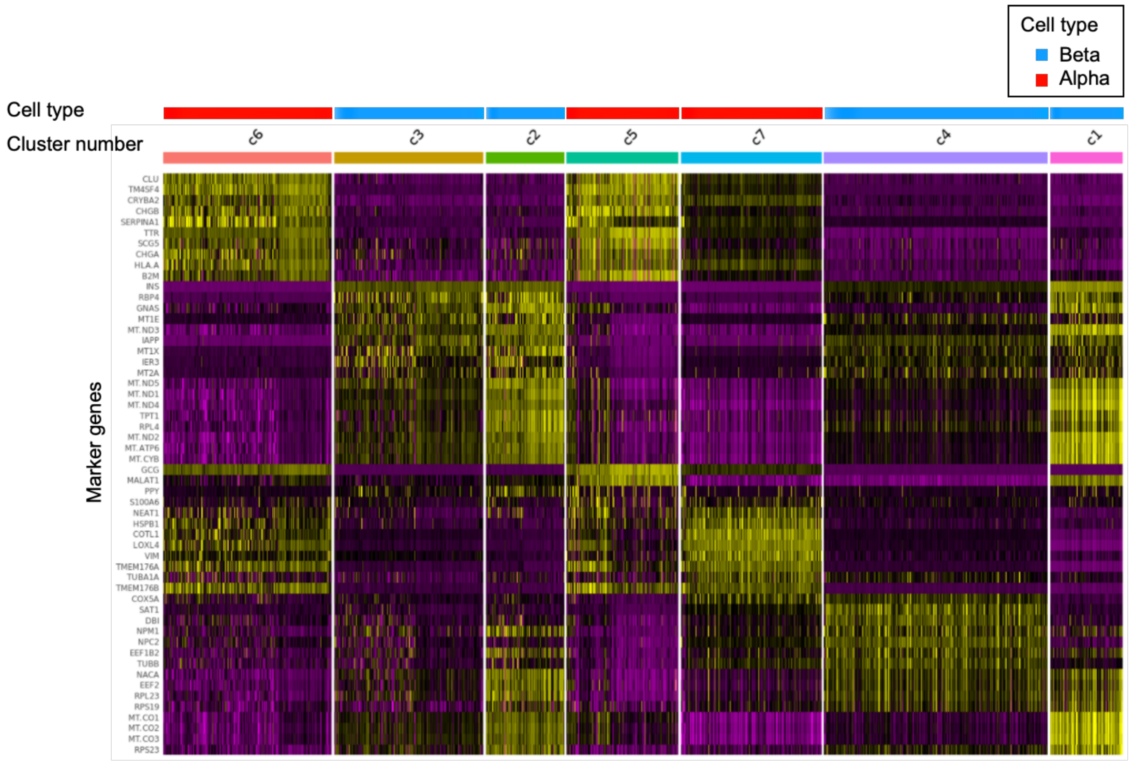

Supplementary Figure 7 | Heatmap of marker genes identified for alpha and beta cells using artemether-induced gene expression data imputed by TIGERS with TT decomposition.

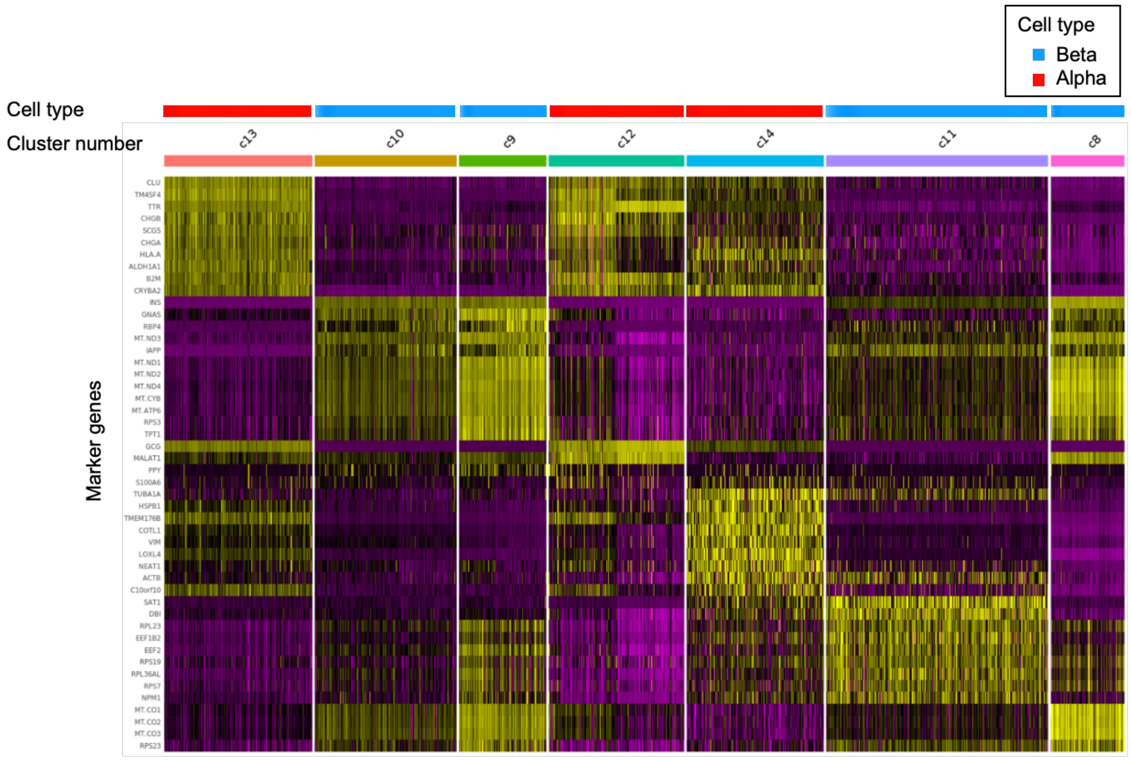

Supplementary Figure 8 | Heatmap of marker genes identified for alpha and beta cells using FoxO-induced gene expression data imputed by TIGERS with TT decomposition.

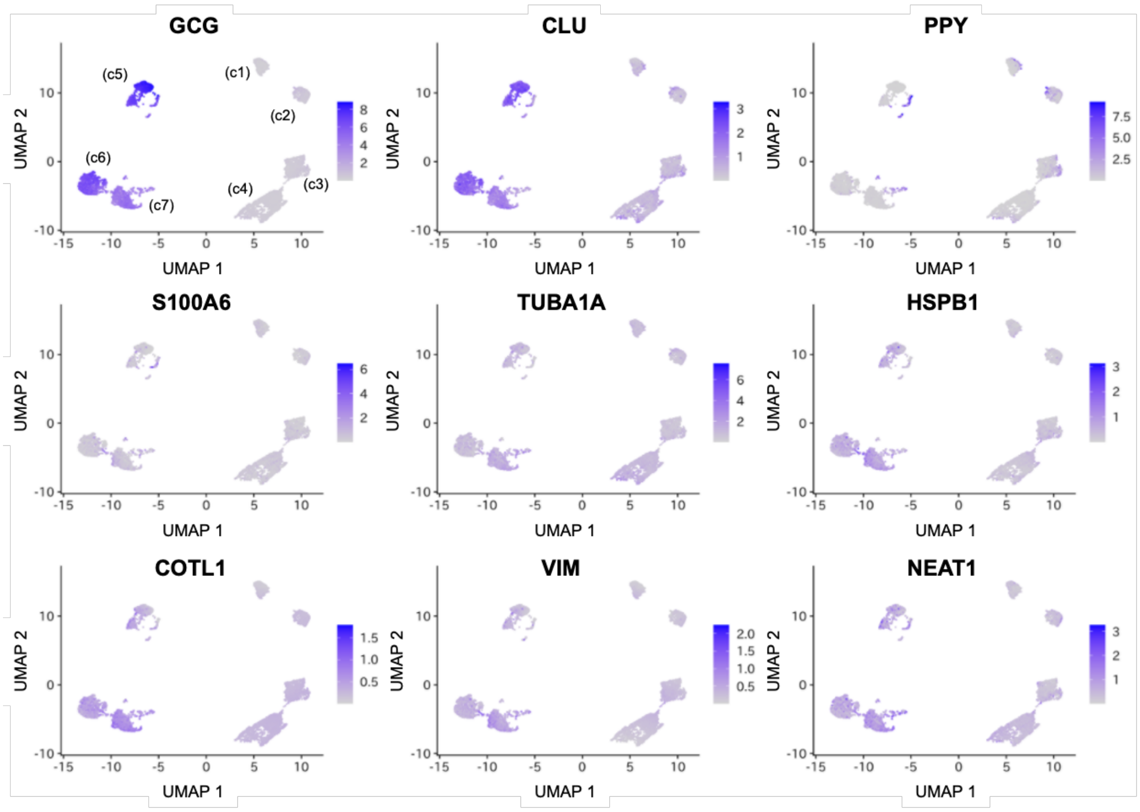

Supplementary Figure 9 | Expression of alpha cell-specific marker genes identified using artemether-induced gene expression data imputed by TIGERS with TT decomposition. The distributions of cells are identical to those in Figure 5b. Each cell is colored according to the expression value of the marker gene. *GCG*, glucagon; CLU, clusterin; *PPY*, pancreatic polypeptide; *S100A6*, S100 calcium binding protein A6; *TUBA1A*, tubulin alpha 1a; *HSPB1*, heat shock protein family B (small) member 1; *COTL1*, coactosin like F-actin binding protein 1; *VIM*, vimentin.

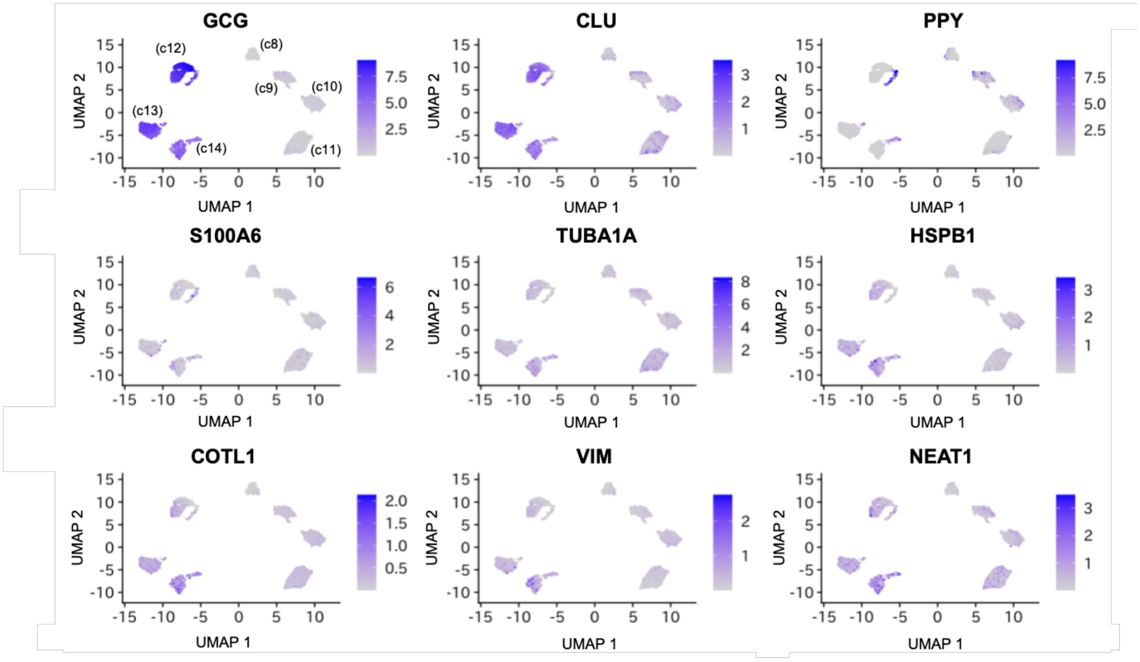

Supplementary Figure 10 | Expression of alpha cell-specific marker genes identified using FoxO-induced gene expression data imputed by TIGERS with TT decomposition. The distributions of cells are identical to those in Figure 5d. Each cell is colored according to the expression value of the marker gene. *GCG*, glucagon; *CLU*, clusterin; *PPY*, pancreatic polypeptide; *S100A6*, S100 calcium binding protein A6; *TUBA1A*, tubulin alpha 1a; *HSPB1*, heat shock protein family B (small) member 1; *COTL1*, coactosin like F-actin binding protein 1; *VIM*, vimentin; *NEAT1*, nuclear paraspeckle assembly transcript 1.

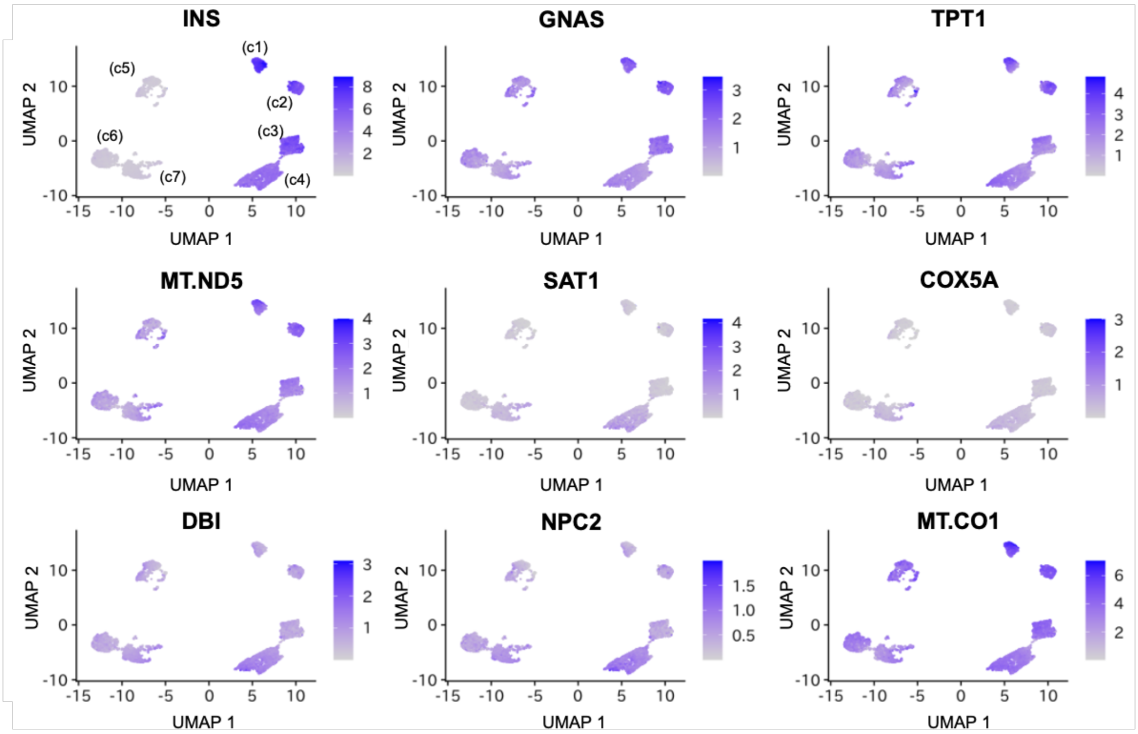

Supplementary Figure 11 | Expression of beta cell-specific marker genes identified using artemether-induced gene expression data imputed by TIGERS with TT decomposition. The distributions of cells are identical to those in Figure 5b. Each cell is colored according to the expression value of the marker gene. *INS*, insulin; *GNAS*, GNAS complex locus; *TPT1*, tumor protein, translationally-controlled 1; *MT.ND5*, mitochondrially encoded NADH:ubiquinone oxidoreductase core subunit 5; *SAT1*, spermidine/spermine N1-acetyltransferase 1; *COX5A*, cytochrome c oxidase subunit 5A; *DBI*, diazepam binding inhibitor, acyl-CoA binding protein; *NPC2*, NPC intracellular cholesterol transporter 2; *MT.CO1*, mitochondrially encoded cytochrome c oxidase I.

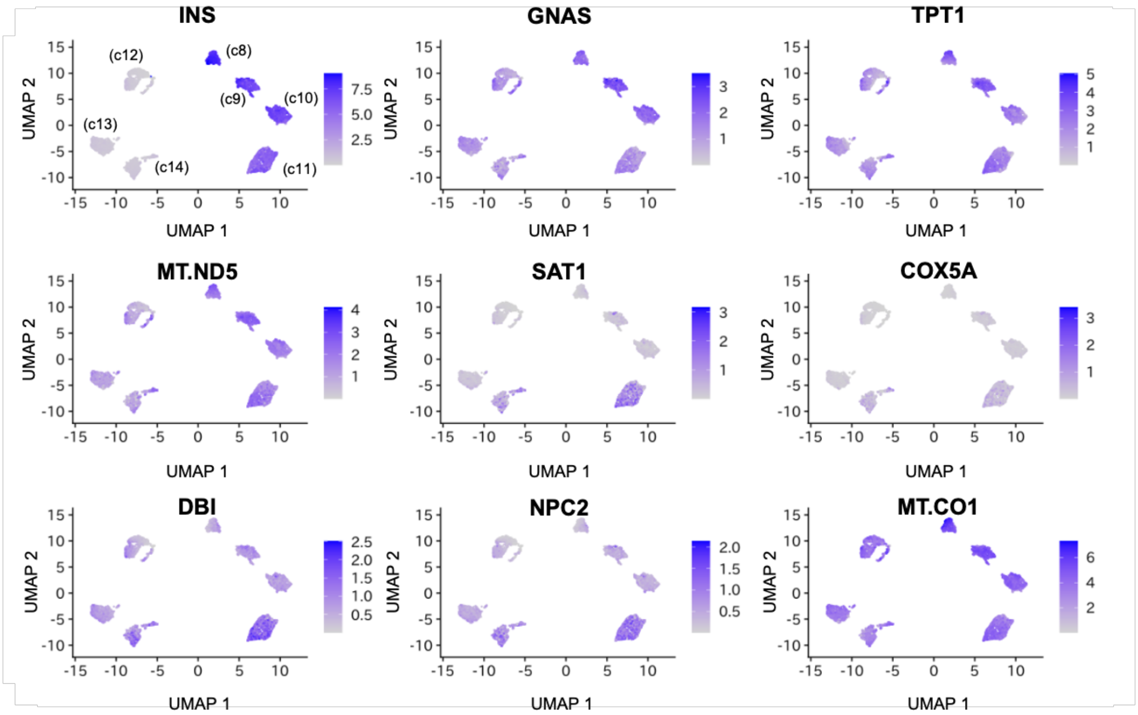

Supplementary Figure 12 | Expression of beta cell-specific marker genes identified using FoxO-induced gene expression data imputed by TIGERS with TT decomposition. The distributions of cells are identical to those in Figure 5d. Each cell is colored according to the expression value of the marker gene. *INS*, insulin; *GNAS*, GNAS complex locus; *TPT1*, tumor protein, translationally-controlled 1; *MT.ND5*, mitochondrially encoded NADH:ubiquinone oxidoreductase core subunit 5; *SAT1*, spermidine/spermine N1-acetyltransferase 1; *COX5A*, cytochrome c oxidase subunit 5A; *DBI*, diazepam binding inhibitor, acyl-CoA binding protein; *NPC2*, NPC intracellular cholesterol transporter 2; *MT.CO1*, mitochondrially encoded cytochrome c oxidase I.

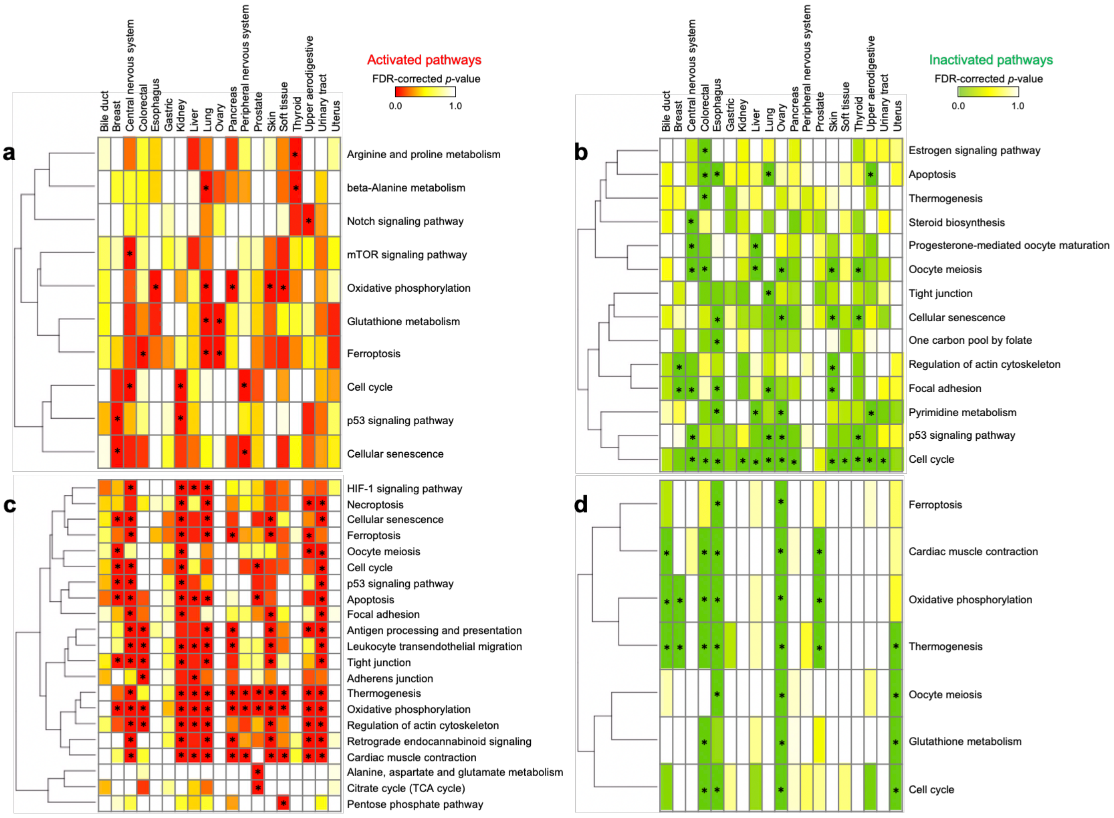

Supplementary Figure 13 | Identification of the mode of action of the anticancer drug afatinib at the single-cell level. **(a)** Activated pathways detected using the unimputed afatinib-induced single-cell gene expression data. **(b)** Inactivated pathways detected using the unimputed afatinib-induced single-cell gene expression data. **(c)** Activated pathways detected using afatinib-induced single-cell gene expression data imputed with TIGERS with TT decomposition. **(d)** Inactivated pathways detected using afatinib -induced single-cell gene expression data imputed with TIGERS with TT decomposition. Pathways are listed according to the complete-linkage clustering on the left of each heatmap. Tissues are listed in the alphabetical order. Colors in the heatmap correspond

to the FDR-corrected p values. Significantly enriched pathways are marked with an asterisk.

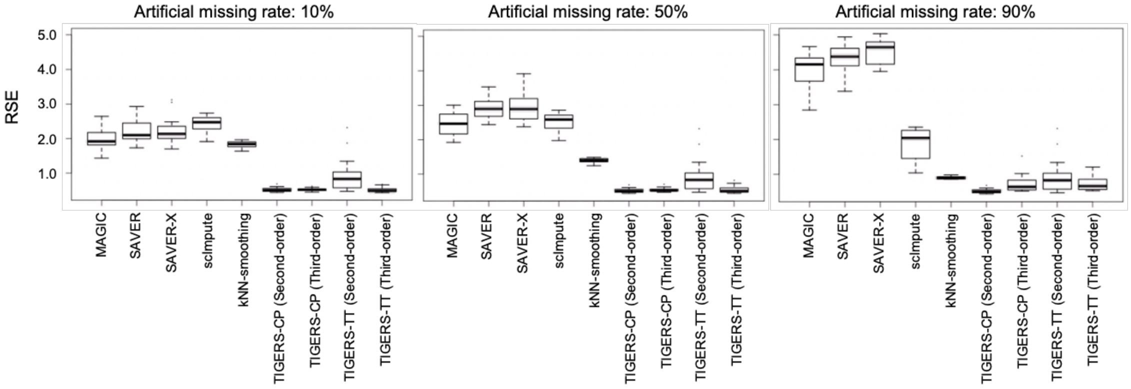

Supplementary Figure 14 | Performance evaluation of data completion in the pancreatic islet dataset between nine imputation methods (*n* = 14 cell types). Except for TIGERS with third-order tensor imputation, all imputation methods are applied to the gene expression matrix. Artificially generated missing rates of 10%, 50%, and 90% were tested. In the box plots: center line, median; box, interquartile range; whiskers, 1.5 × interquartile range; dots, outliers.

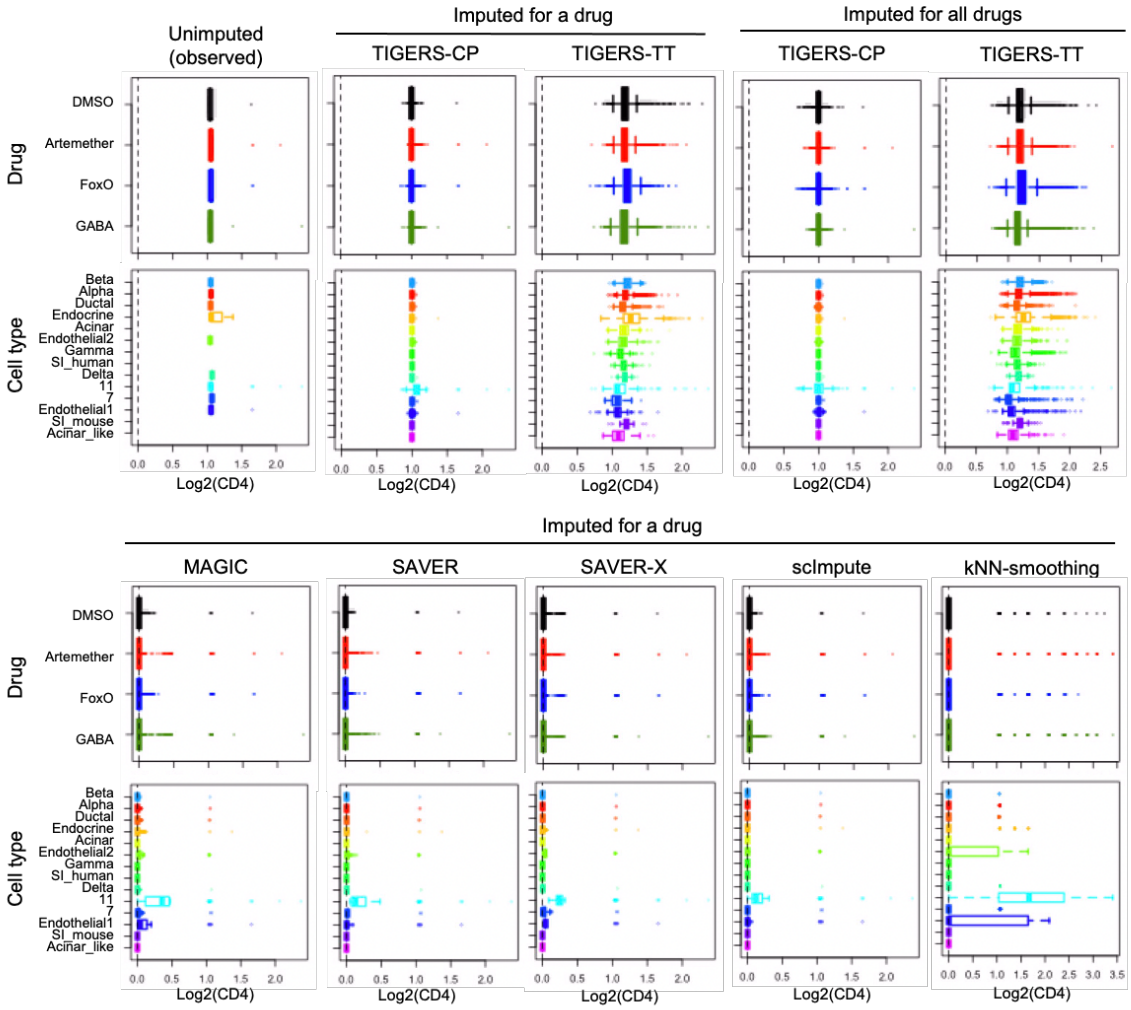

Supplementary Figure 15 | Distribution of log2 expression of CD4 with and without imputation. A pseudo count (i.e., 1.0) was added to CD4 expression prior to log2 transformation. For cells treated by a drug and those imputed for all drugs, 14,368 cells, each treated by a single drug, and 57,472 (= 14,368 cells × 4 drugs) profiles were evaluated, respectively. In the box plots: center line, median; box, interquartile range; whiskers, 1.5 × interquartile range; dots, outliers.

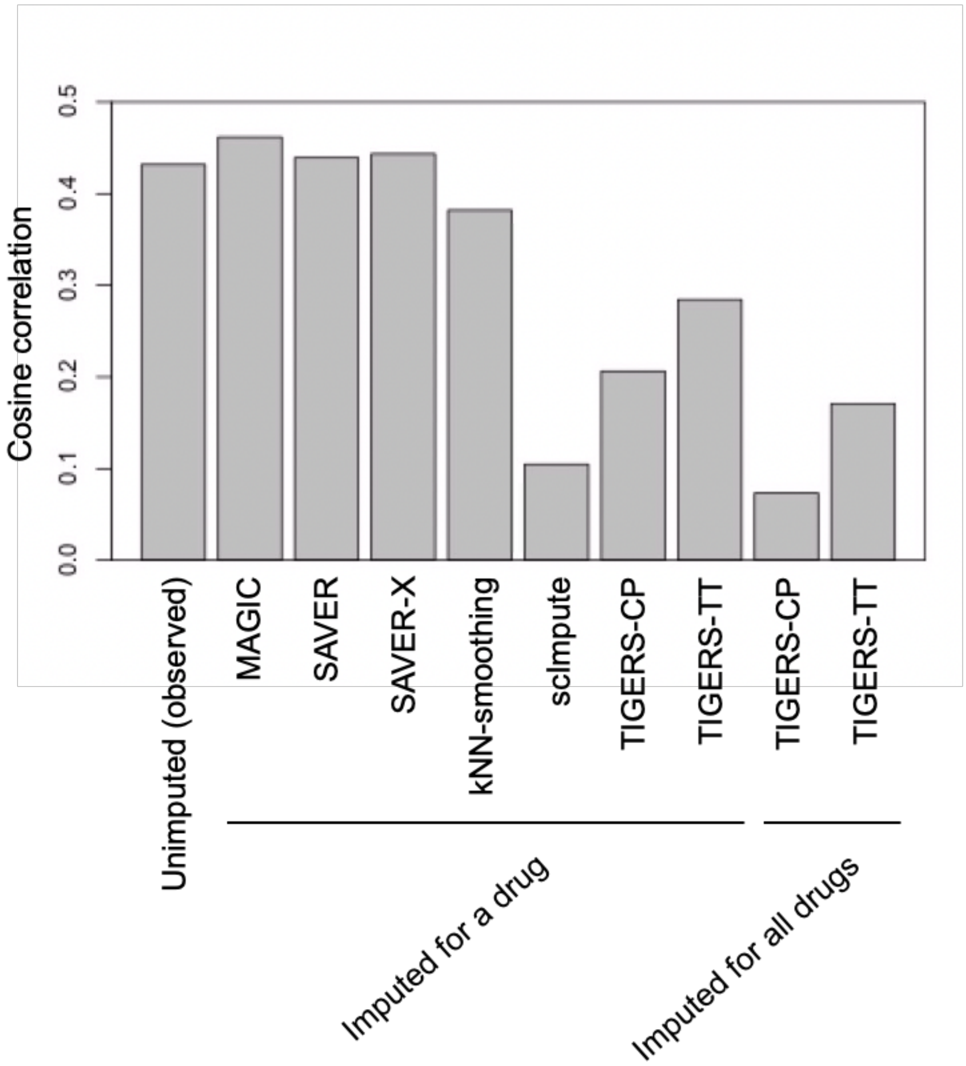

Supplementary Figure 16 | Correlations between bulk RNA-seq and single-cell RNA-seq dataset with and without imputation. For cells treated by a drug and those imputed for all drugs, 14,368 cells, each treated by a single drug, and 57,472 (= 14,368 cells × 4 drugs) profiles were evaluated, respectively.

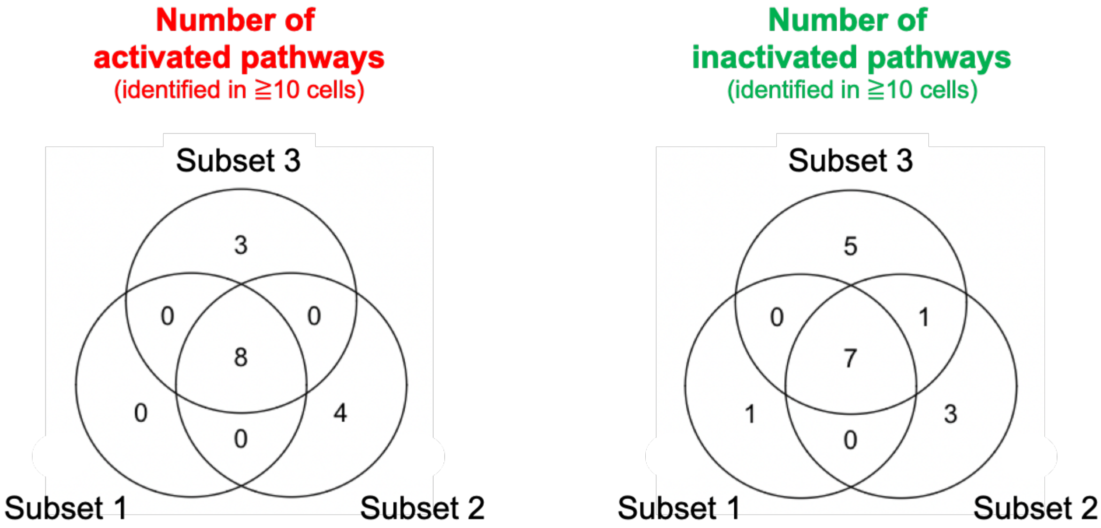

Supplementary Figure 17 | Comparisons of the numbers of activated and inactivated pathways for artemether-induced gene expression signatures constructed using the subsetted datasets of gamma cells in the pancreatic dataset. All pathways were detected at a significance level of $p < 0.05$.

Supplementary References

1. Lamb, J. *et al.* The Connectivity Map: using gene-expression signatures to connect small molecules, genes, and disease. *Science* **313**, 1929–1935 (2006).

2. Subramanian, A. *et al.* A next generation connectivity map: L1000 platform and the first 1,000,000 profiles. *Cell* **171**, 1437–1452 (2017).

3. Marquina-Sanchez, B. *et al.* Single-cell RNA-seq with spike-in cells enables accurate quantification of cell-specific drug effects in pancreatic islets. *Genome Biol* **21**, 1–22 (2020).

4. Hao, Y. *et al.* Integrated analysis of multimodal single-cell data. *Cell* (2021).
