# Supplementary material for: Pathway trajectory analysis with tensor imputation reveals drug-induced single-cell transcriptomic landscape
Source: Nat Comput Sci. 2022 Nov 24;2(11):758–70. doi: 10.1038/s43588-022-00352-8 (PMC10768635; doi:10.1038/s43588-022-00352-8)
Supplement: Supplementary file 2 — Reporting Summary [file 43588_2022_352_MOESM2_ESM.pdf]

nature portfolio

Corresponding author(s): Yoshihiro Yamanishi

Last updated by author(s): Oct 7, 2022

Reporting Summary

Nature Portfolio wishes to improve the reproducibility of the work that we publish. This form provides structure for consistency and transparency in reporting. For further information on Nature Portfolio policies, see our Editorial Policies and the Editorial Policy Checklist.

Statistics

For all statistical analyses, confirm that the following items are present in the figure legend, table legend, main text, or Methods section.

n/a	Confirmed	
☐	☒	The exact sample size (*n*) for each experimental group/condition, given as a discrete number and unit of measurement
☒	☐	A statement on whether measurements were taken from distinct samples or whether the same sample was measured repeatedly
☐	☒	The statistical test(s) used AND whether they are one- or two-sided<br>*Only common tests should be described solely by name; describe more complex techniques in the Methods section.*
☒	☐	A description of all covariates tested
☐	☒	A description of any assumptions or corrections, such as tests of normality and adjustment for multiple comparisons
☐	☒	A full description of the statistical parameters including central tendency (e.g. means) or other basic estimates (e.g. regression coefficient) AND variation (e.g. standard deviation) or associated estimates of uncertainty (e.g. confidence intervals)
☐	☒	For null hypothesis testing, the test statistic (e.g. *F*, *t*, *r*) with confidence intervals, effect sizes, degrees of freedom and *P* value noted<br>*Give P values as exact values whenever suitable.*
☒	☐	For Bayesian analysis, information on the choice of priors and Markov chain Monte Carlo settings
☒	☐	For hierarchical and complex designs, identification of the appropriate level for tests and full reporting of outcomes
☒	☐	Estimates of effect sizes (e.g. Cohen's *d*, Pearson's *r*), indicating how they were calculated

Our web collection on statistics for biologists contains articles on many of the points above.

Software and code

Policy information about availability of computer code

Data collection	Cell Ranger software, Seurat package (v.3.1.5), Monocle3 package, DESeq package (v.2)
Data analysis	Standard imputations were executed using the Rmagic (version 2.0.3), SAVER (version 1.1.2), SAVER-X (version 1.0.2), and scImpute (version 0.0.9) packages in R (version 4.1.3) and kNN-smoothing algorithm (version 2.1) in Python (version 3.7.13). The code for tensor imputations (TIGERS) has been provided in a Code Ocean link (https://doi.org/10.24433/CO.7383485.v1).

For manuscripts utilizing custom algorithms or software that are central to the research but not yet described in published literature, software must be made available to editors and reviewers. We strongly encourage code deposition in a community repository (e.g. GitHub). See the Nature Portfolio guidelines for submitting code & software for further information.

Data

Policy information about availability of data

All manuscripts must include a data availability statement. This statement should provide the following information, where applicable:

- Accession codes, unique identifiers, or web links for publicly available datasets
- A description of any restrictions on data availability
- For clinical datasets or third party data, please ensure that the statement adheres to our policy

All data are available from public repositories. The pancreatic islet dataset was downloaded from the NCBI GEO (accession number, GSE142465). The cancer cell dataset was obtained from figshare at https://figshare.com/s/139f64b495dea9d88c70. Drug-induced bulk gene expression data was downloaded from the NCBI

GEO (accession numbers, GSE70138 and GSE92742). The coupled RNA-seq and scRNA-seq datasets were downloaded from the NCBI GEO (accession numbers, GSE148465 and GSE149214, respectively). A subset of the data is available from Code Ocean (https://doi.org/10.24433/CO.7383485.v1). Source Data for Figures 2, 3, and 5 is available with this manuscript. Other Source Data is available on figshare (https://doi.org/10.6084/m9.figshare.21222047).

Human research participants

Policy information about studies involving human research participants and Sex and Gender in Research.

Reporting on sex and gender	n/a
Population characteristics	n/a
Recruitment	n/a
Ethics oversight	n/a

Note that full information on the approval of the study protocol must also be provided in the manuscript.

Field-specific reporting

Please select the one below that is the best fit for your research. If you are not sure, read the appropriate sections before making your selection.

☒ Life sciences ☐ Behavioural & social sciences ☐ Ecological, evolutionary & environmental sciences

For a reference copy of the document with all sections, see nature.com/documents/nr-reporting-summary-flat.pdf

Life sciences study design

All studies must disclose on these points even when the disclosure is negative.

Sample size	No sample size calculation was performed for this study. For the pancreatic islet dataset, we extracted gene expression profiles in pancreatic islets from a human donor and four perturbations, namely, DMSO, artemether, the FoxO inhibitor AS1842856 (FoxOi), and GABA, which were measured within 72 h after treatment. In total, 14,368 cells were obtained from this dataset. For the cancer cell dataset, We extracted gene expression profiles in an experiment (experiment number 10), where nine perturbations, namely, DMSO, everolimus, afatinib, taselisib, AZD5591, JQ1, gemcitabine, trametinib, and prexasertib were administered, and gene expression was measured within 24 h. In total, 31,438 cells were used from this dataset.
Data exclusions	For the pancreatic islet dataset, we removed cells for which empty droplets or potential doublets were suspected in the information on the downloaded annotation file.
Replication	We assessed our findings on different datasets. All replication attempts were successful.
Randomization	In the performance evaluation of the data imputation task, we randomly added artificial missing values to the observed data and tested whether the tensor imputation algorithms could correctly recover these values.
Blinding	Our study is not a clinical study. Thus, group allocation during data collection and analysis are out of scope.

Reporting for specific materials, systems and methods

We require information from authors about some types of materials, experimental systems and methods used in many studies. Here, indicate whether each material, system or method listed is relevant to your study. If you are not sure if a list item applies to your research, read the appropriate section before selecting a response.

Materials & experimental systems

n/a	Involved in the study	
☒	☐	Antibodies
☒	☐	Eukaryotic cell lines
☒	☐	Palaeontology and archaeology
☒	☐	Animals and other organisms
☒	☐	Clinical data
☒	☐	Dual use research of concern

Methods

n/a	Involved in the study	
☒	☐	ChIP-seq
☒	☐	Flow cytometry
☒	☐	MRI-based neuroimaging
